# Unraveling the Role of Interfaces on the Spall Failure of Cu/Ta Multilayered Systems

**DOI:** 10.1038/s41598-019-57048-9

**Published:** 2020-01-14

**Authors:** Jie Chen, Suveen N. Mathaudhu, Naresh Thadhani, Avinash M. Dongare

**Affiliations:** 1 Department of Materials Science and Engineering, and Institute of Materials Science, University of Connecticut, Storrs, CT 06269 USA; 2 Mechanical Engineering, and Materials Science and Engineering, University of California, Riverside, CA 92507 USA; 3 Department of Materials Science and Engineering, Georgia Institute of Technology, Atlanta, GA 30332 USA

**Keywords:** Metals and alloys, Atomistic models

## Abstract

Molecular dynamics (MD) simulations are carried out to investigate the effects of the type and spacing of FCC/BCC interfaces on the deformation and spall behavior. The simulations are carried out using model Cu/Ta multilayers with six different types of interfaces. The results suggest that interface type can significantly affect the structure and intensity of the incoming shock wave, change the activated slip systems, alter dislocation slip and twinning behavior, affect where and how voids are nucleated during spallation and the resulting spall strength. Moreover, the above aspects are significantly affected by the interface spacing. A transition from homogeneous to heterogeneous dislocation nucleation occurs as the interface spacing is decreased to 6 nm. Depending on interface type and spacing, damage (voids) nucleation and spall failure is observed to occur not only at the Cu/Ta interfaces, but also in the weaker Cu layer interior, or even in the stronger Ta layer interior, although different mechanisms underlie each of these three distinct failure modes. These findings point to the fact that, depending on the combination of interface type and spacing, interfaces can lead to both strengthening and weakening of the Cu/Ta multilayered microstructures.

## Introduction

Nanoscale multilayered materials are an emerging class of materials that render a unique combination of high thermal stability, strength, and damage resistance^1,2^. This unique response is due to a high density of interfaces in the microstructure that can be used to tailor their performance. Multilayer microstructures, therefore, are very promising materials for next-generation damage-tolerant applications. Recent advancements in experimental capabilities based on accumulative roll bonding (ARB)^3^, and physical vapor deposition (PVD)^4^ has enabled the fabrication of a wide range of novel FCC/BCC bi-metallic multilayered microstructures. Such capabilities open up opportunities to tailor the interface structure and spacing in designing damage-resistant microstructures.

A substantial amount of research, therefore, aims to understand the role of interfaces on the mechanical behavior of nanoscale multilayers^5–12^. Like grain boundaries in the nanocrystalline metals, bi-metallic interfaces are expected to result in significant differences in the deformation mechanisms of the multilayered microstructure as compared to their single-phase counterparts, by acting as sources or sinks for dislocation nucleation, or as barriers to dislocation propagation^13^. The capability of the bi-metallic interfaces to hinder dislocation propagation critically determines the strength of the multilayers^7,14^. This capability is also affected by the local atomic structure of the interface. For example, Zheng *et al*.^15^ showed that for Cu/Nb multilayers, a faceted interface such as the KS112 interface could significantly promote twinning, due to the presence of atomic steps and the associated misfit dislocations with out-of-plane Burgers’ vector that act as sources for nucleating twinning partials. Moreover, the overall deformation modes and the resulting strengths of the multilayers are length-scale (spacing between the interface) dependent^16–20^.

Recent studies also aim to understand the role of interfaces in modifying the deformation and failure behavior of individual phases under extreme environments of shock loading conditions. The shock compression behavior (dislocation slip vs deformation twinning) and spall failure (void nucleation and growth) behavior of multilayered microstructures are observed to vary with interface structure and spacing. For example, shock response (post-shock microstructure) of PVD-synthesized Cu-Nb composites suggests increased dislocation densities and deformation twinning in the Cu layers^21^, whereas no such response is observed for the ARB-synthesized Cu-Nb multilayered composites^22^ under similar loading conditions (shock stress). This difference in behavior is likely due to the difference in interface morphologies (typically flat for PVD vs faceted for ARB) as well as the layer thickness. Also, while one may expect that the distribution of interfaces will dominate the spall failure behavior of these microstructures, voids are observed to nucleated inside the Cu layers in addition to nucleation at the Cu/Nb interfaces^21^. However, a systematic study of the role of length scales (spacing) of interfaces on the deformation and spall failure behavior is currently missing. For example, it is not clear if a decrease in layer spacing will modify the deformation twinning/de-twining behavior of not only the Cu layers but also the Ta layers. Also, this raises the questions: (a) *Will the spall failure always be observed in the weaker Cu layers (interior and near interfaces), or is this determined by the structure and spacing of the interfaces wherein voids can be observed to nucleate and grow in the Ta layers?* (b) *Does the observed length scale dependence of spall strength values for the multilayered structures also exist for all interface structures?* While the current state-of-art experimental capabilities use *in situ* femtosecond XRD for characterization of deformation mechanisms under shock loading conditions^23–25^, such questions are still very challenging to explore experimentally, given both the small length scales of the phenomena and the short time scales over which they occur.

Molecular dynamics (MD) simulations can provide atomic-scale resolution of the processes occurring under dynamic loading conditions, thus enabling us to unravel the microstructural features contributing to the shock response and spall failure behavior^26–32^. While MD simulations have provided valuable insights on the role of interface structure and spacing on the defect (dislocations, twins) nucleation, evolution and transmission behavior in multilayered microstructures^33–39^, the understanding of the shock response and spall failure behavior is still in infancy. For the case of FCC microstructures, the density of twinning partials and stair-rod partials in the microstructure determines the predicted spall strength values^29,40,41^. Whereas for the case of Cu microstructures, the lowest density of stair-rod partials is also associated with the highest density of twinning partials at the spall plane and renders higher spall strength values^40^. For multilayered FCC/BCC microstructures, the current studies investigating the shock response have largely focused on the wave propagation behavior^42,43^, dislocation nucleation behavior^44^ and spall failure^45^ in Cu/Nb composites. These studies suggest that the layer spacing results in the modifications of the dislocation nucleation behavior (homogeneous vs heterogeneous) in only the Cu layers (with Nb layers showing always homogeneous nucleation of dislocations)^44^, and the voids nucleate in the Cu layers near the Cu/Nb interface^45^. The recent study on Cu/Ta layers work has investigated the role of interface spacing for Cu/Ta multilayers with the KS interface, wherein interface spacing is found to determine the failure modes and a strong correlation between the dislocation density and spall strength values is demonstrated. The study suggests that the spall strength varies with dislocation density in the Cu phase as voids are observed to nucleate in the weaker Cu phase. A high values of spall strength is observed for the microstructure that has the higher density of Shockley partials, stair-rod partials, and twinning partials. The Cu/Ta multilayered microstructures with an interface spacing greater than 6 nm render spall strengths that are higher than pure Cu. No voids are observed to nucleate in the Ta phase for layer spacings ranging from 3 nm to 47 nm for the KS interfaces^46^. However, it is not clear if this length scale and dislocation density dependence of spall strengths observed for KS interfaces is consistent for all interface structures. While the Ta layers do not show failure for KS Cu/Ta interfaces, it is not clear if the interface structure plays a role in determining the deformation behavior of Ta that will result in spall failure in the Ta layers. Does failure always occur at the interface or the weaker phase? Such understanding is missing due to a lack of systematic study.

This work, therefore, aims to carry out a systematic study of the role of the structure (orientation) and spacing of Cu/Ta FCC/BCC interfaces on the atomic scale deformation mechanisms during shock compression and spall behavior of the multilayered microstructures using MD simulations. The study uses six Cu/Ta multilayered microstructures with six commonly occurring FCC/BCC interfaces with layer spacing ranging from 6 nm to 47 nm for each layer as model systems.

## Structure and Properties of Model Cu/Ta Interfaces

**Figure 1:**
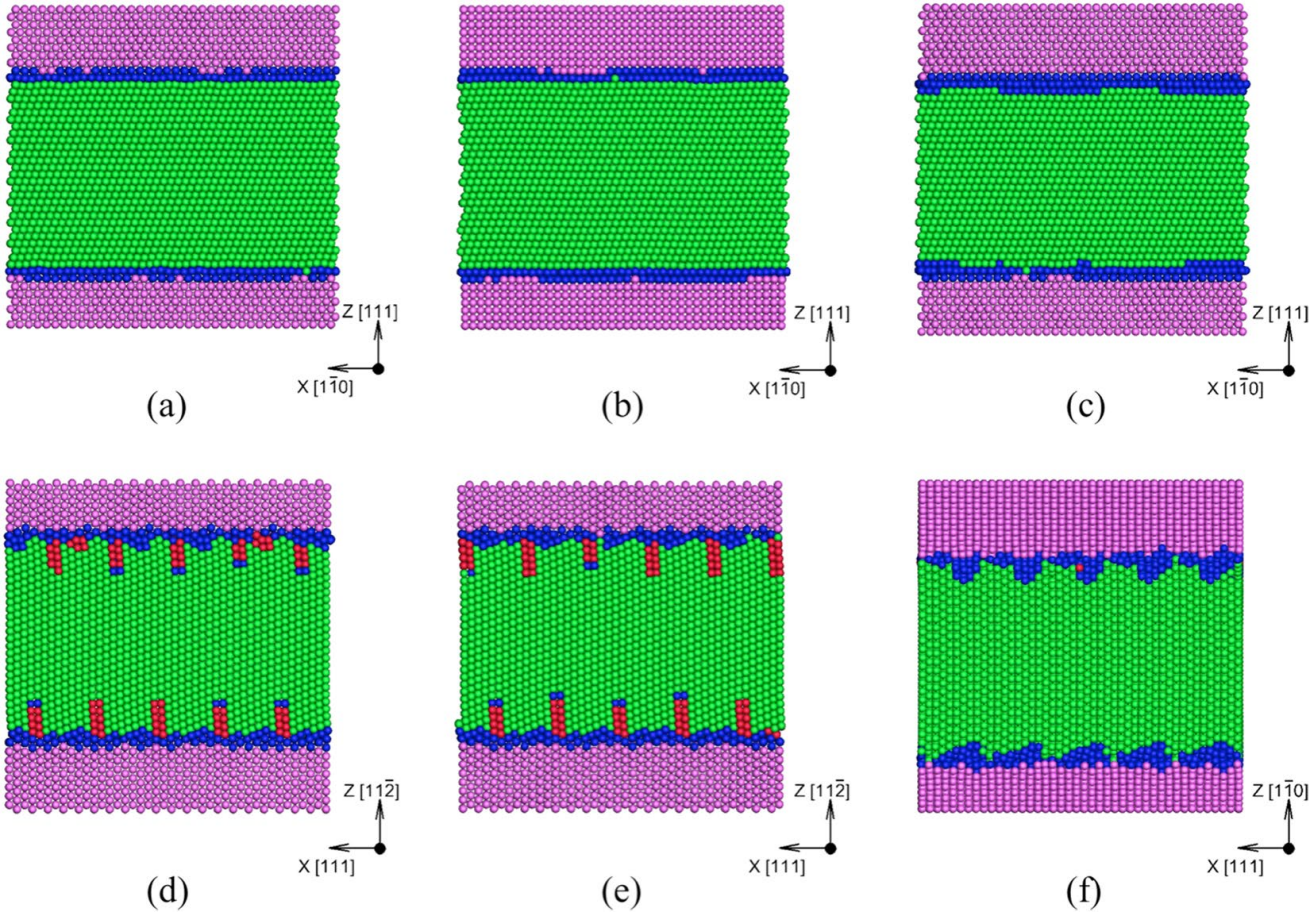
Side view of the relaxed atomic structures of the model Cu/Ta interfaces: (**a**) KS, (**b**) NW, (**c**) KS_2_, (**d**) KS112-case1, (**e**) KS112-case2, (f) OT. Atoms are colored in the following way: Cu FCC stacking (green), Cu HCP stacking (red), Ta BCC stacking (purple) and disordered (blue).

Six model Cu/Ta interfaces are chosen to study the effects of the interface type and spacing on spall behavior. The selected interfaces include the most commonly occurring interfaces in the experimental study of FCC/BCC multilayers. These are (orientation relationships in brackets): Kurdjumov–Sachs “KS”, (FCC (111) || BCC (1$$\overline{1}$$0), FCC [1$$\overline{1}$$0] || BCC [111]); Nishiyama–Wassermann “NW”, (FCC (111) || BCC (1$$\overline{1}$$0), FCC [1$$\overline{1}$$0] || BCC [001]); the “KS112” (FCC (11$$\overline{2}$$) || BCC (11$$\overline{2}$$), FCC [1$$\overline{1}$$0] || BCC [111])^22,47–52^; the “KS_2_” $$(FCC(111)||{\rm{B}}{\rm{C}}{\rm{C}}\,(1\bar{1}0)$$$$,\,FCC[1\bar{1}0]||BCC[111])$$^53^; and the “OT” (other for simplicity), (FCC (1$$\overline{1}$$0) || BCC (001), FCC [111] || BCC [1$$\overline{1}$$0])^48^ interface. For the KS112 interface, two variants are constructed, depending on the combination of the number of Cu and Ta planes joined normal to the interface, denoted as the KS112-case1 and KS112-case2 here. The as-created structures are minimized to identify the type of the interface (flat vs faceted), the defect structures and to calculate the interface energy. The energies of the six interfaces are tabulated in Table 1 and the relaxed initial structures (side views) are shown in Figure 1. The images show that the KS, NW, and KS_2_ are flat interfaces, with in-plane misfit dislocations, whereas KS112-case1, KS112-case2, and OT are faceted ones, with partial dislocations extending into the Cu lattice. While in-plane misfit dislocation patterns are observed to be the same for the two KS112 variants, the out-of-plane misfit dislocation patterns are different. The position of the stacking fault planes extending into the Cu lattice at the top Cu/Ta interface is shifted to the right by two atomic planes for the KS112-case2 as compared to the KS112-case1 interface (Figure 1(d), and (e)). Among the flat interfaces, KS and NW interfaces display similar interface energy, whereas the KS_2_ interface displays slightly higher interface energy values attributed to the rearrangement of the Cu layer adjacent to the interface Ta layer^53^. Faceted interfaces have higher interface energies than the flat ones with the highest value calculated for the OT interface. In addition, these interfaces show significant variations in the type and density of pre-existing dislocations, including Shockley partials, stair-rod partials, and twinning partials. No pre-existing dislocations of these types are identified for the flat interfaces, whereas a high density of Shockley partials exists for both the KS112 interfaces and Shockley partials and twinning partials for the OT interface. Such variations are expected to contribute to variations in the shock and spall responses of the Cu/Ta multilayers. The densities of pre-existing dislocations per Cu/Ta interface in the multilayered microstructure are provided in Supplementary Note 1.

**Table 1:** Model Cu/Ta interfaces considered in this work, and their type (flat/faceted), orientation relationship and interface energy (γ).

Interface	Type	Orientation relationship	γ (mJ/m^2^)
KS	Flat	(111) 〈1$\overline{1}$0〉 Cu || (1$\overline{1}$0) 〈111〉 Ta	206.97 ± 10
NW	Flat	(111) 〈1$\overline{1}$0〉 Cu || (1$\overline{1}$0) 〈001〉 Ta	197.81 ± 10
KS_2_	Flat	(111) 〈1$\overline{1}$0〉 Cu || (1$\overline{1}$0) 〈111〉 Ta	225.29 ± 10
KS112-case1	Faceted	(11$\overline{2}$) 〈111〉 Cu || (11$\overline{2}$) 〈1$\overline{1}$0〉 Ta	608.62 ± 10
KS112-case2	Faceted	(11$\overline{2}$) 〈111〉 Cu || (11$\overline{2}$) 〈1$\overline{1}$0〉 Ta	608.63 ± 10
OT	Faceted	(1$\overline{1}$0) 〈111〉 Cu || (001) 〈1$\overline{1}$0〉 Ta	900.41 ± 10

The six Cu/Ta interface structures considered not only allow investigation of the role of type (flat vs faceted) but also enable investigation of the role of loading orientations (the Z direction of Cu and Ta layers) on the shock compression and spall failure response. The three loading orientations considered are: Cu (111) || Ta (1$\overline{1}$0) for the flat interfaces (KS, NW and KS_2_), Cu (11$\overline{2}$) || Ta (11$\overline{2}$) for both the KS112 interfaces and Cu (1$\overline{1}$0) || Ta (001) for the OT interface. All three flat interfaces and the two variants of KS112 interface share the same loading orientation but a different local interface structure.

**Figure 2:**
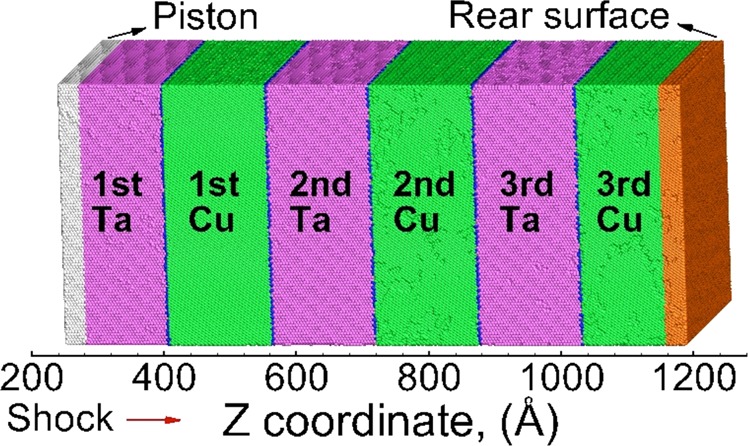
Initial setup of the shock simulation, for Cu/Ta multilayers with the KS interface at an interface spacing of 16 nm. During the shock loading, the piston atoms (silver) are driven inward (positive Z direction) at 1 km/s for 10 ps (red arrow). The Cu/Ta interfaces are shown by the blue atoms (disordered). Starting from the leftmost Ta layer, the consecutive Ta and Cu layers are labeled as the 1st Ta layer, 1st Cu layer, 2nd Ta layer, 2nd Cu layer, … etc.

This manuscript investigates the shock and spall failure behavior of the Cu/Ta multilayered microstructures with the orientation relationships and interface spacings listed above. Figure 2 shows an example of a Cu/Ta multilayered initial microstructure with the KS interface between Cu and Ta. Here, Cu atoms colored as green for FCC stacking, red for HCP stacking, and blue for disordered. Similarly, the Ta atoms colored as purple for BCC stacking and blue for disordered. The Cu/Ta multilayered microstructures contain ~ 10 million atoms, with a dimension of ~ 40 nm x ~ 40 nm x ~ 100 nm. The effects of interface structure and spacing on the observed deformation and failure mechanisms are investigated and discussed below.

## Results and Discussion

### Shock deformation and spall behavior of Cu/Ta multilayers

The spall behavior of Cu/Ta multilayers is first investigated for a fixed interface spacing of 16 nm. This interface spacing is chosen since it has been shown to lead to a very high spall strength in the previous study^46^. The entire simulation duration is divided into four stages and marked here for analysis: Stage I (SI) – shockwave compression generated by driving the piston for a given pulse (10 ps); Stage II (SII) – release of the compression wave starting at 10 ps (end of pulse) and ends when the compression wave reaches the rear surface at ~20 ps; Stage III (SIII) – starts at the expansion of the rear surface, interaction of release waves and creation of triaxial tensile pressures that results in onset of spall failure (void nucleation); and Stage IV (SIV) – corresponds to void nucleation, growth and spallation. The shock response of the Cu/Ta multilayers will be discussed in detail for all four stages.

**Figure 3:**
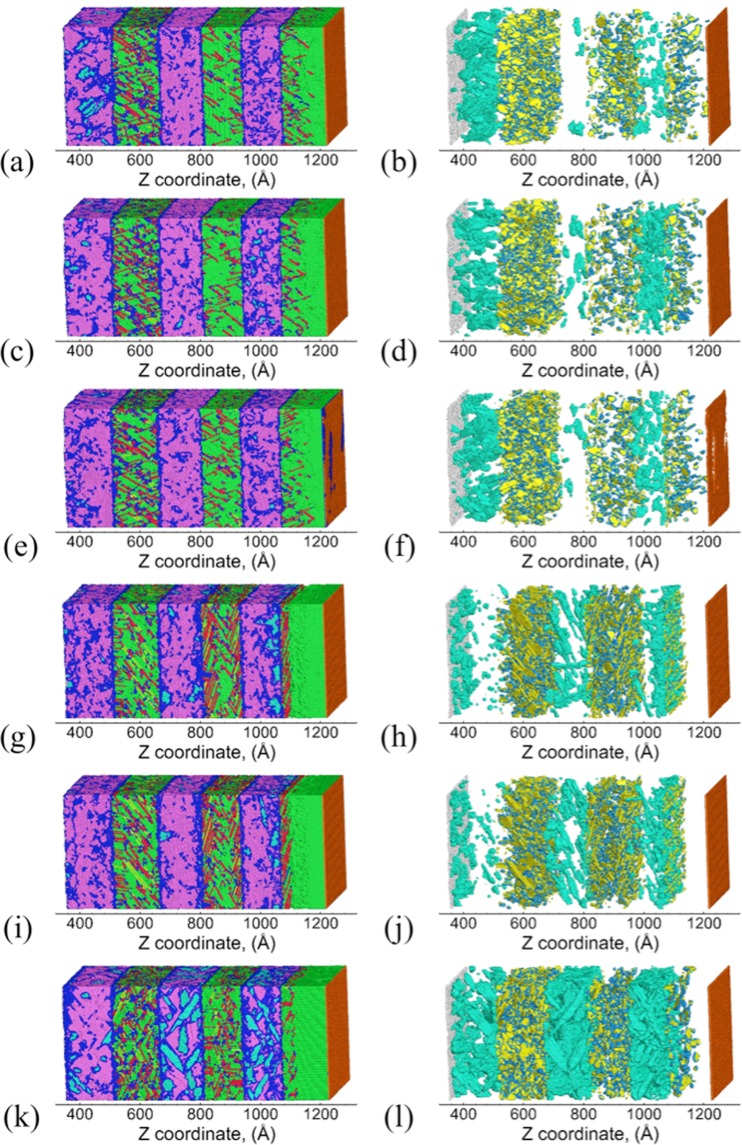
Snapshots of Cu/Ta multilayers at an interface spacing of 16 nm at the end of SII (~20 ps): (**a**,**b**) KS, (**c**,**d**) NW, (**e**,**f**) KS_2_, (**g**,**h**) KS112-case1, (**i**,**j**) KS112-case2, (**k**,**l**) OT. Right-hand panels show the distribution of defects (Cu twin faults, Cu twinning partials, and Ta twin faults) and damage (Cu surfaces and Ta surfaces). Atoms are colored differently: green for FCC Cu, red for Cu stacking faults, yellow for Cu twin faults, light blue for Cu twinning partials, orange for Cu surface/voids, purple for BCC Ta, cyan for Ta twin faults, silver for Ta surface/voids, and blue for disordered Cu or Ta atoms.

**Figure 4:**
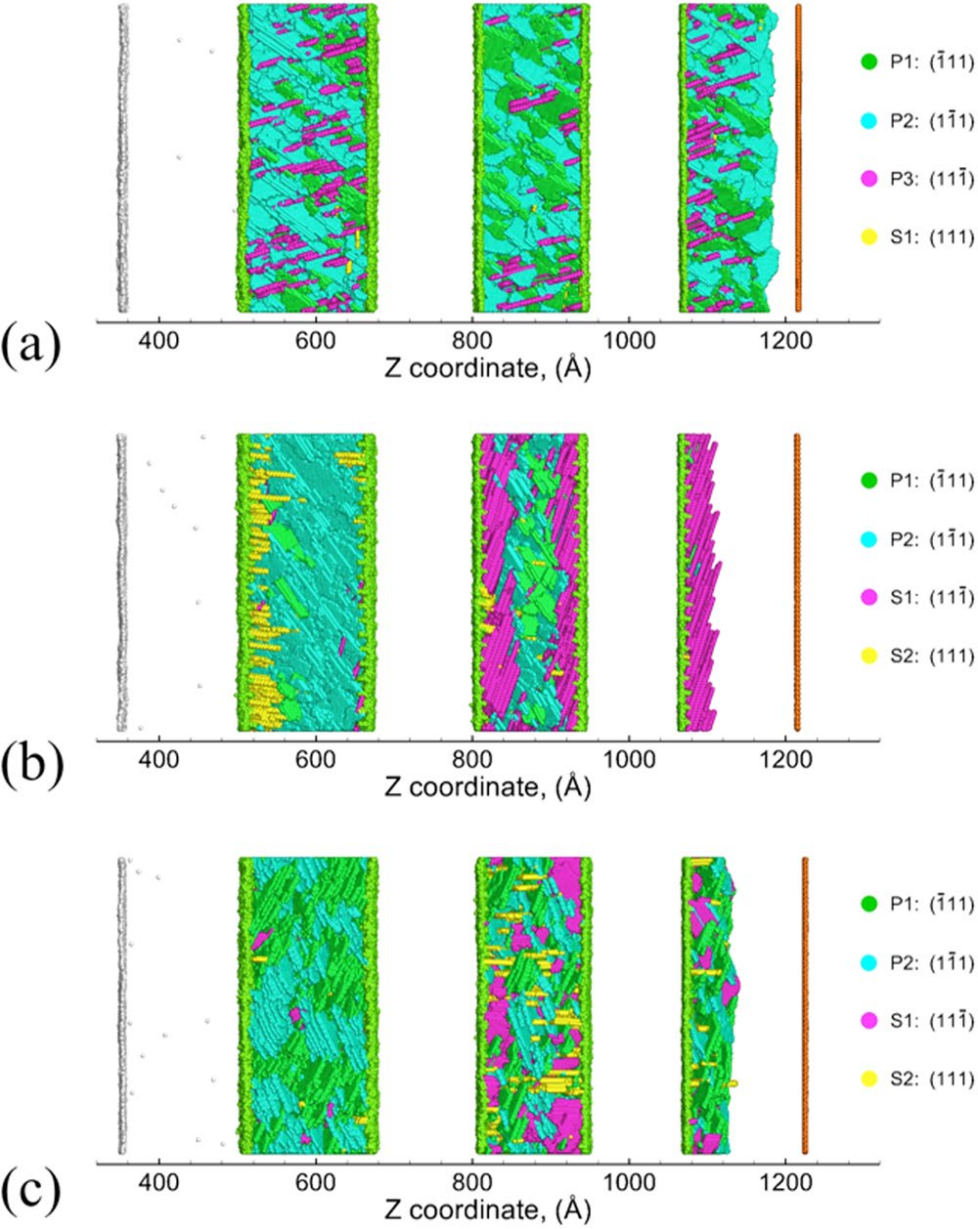
Snapshots showing the activated slip planes in Cu/Ta multilayers at an interface spacing of 16 nm at the end of SII (~20 ps): (**a**) KS, (**b**) KS112-case1, (**c**) OT. Only atoms corresponding to the slip planes in the Cu layers (Cu stacking/twin faults) and damage (Cu surfaces and Ta surfaces) are shown. In the Cu layers atoms are colored based on the slip planes they correspond to, as shown on the right-hand side. Atoms corresponding to the Cu/Ta interfaces are shown as light green.

Figure 3 shows the snapshots of the Cu/Ta multilayers and the corresponding defect microstructures at the end of SII. For all the flat interfaces, stacking/twin faults are nucleated along three primary slip planes in Cu layers: P1-($\overline{1}$11), P2-(1$\overline{1}$1), P3-(11$\overline{1}$), although a few stacking/twin faults are also observed along the secondary slip plane S1-(111). Figure 4(a) shows these activated slip planes for the KS interface, wherein atoms are colored based on the slip planes they correspond to in the Cu layers. The three given primary slip planes lie at 70.53° with respect to the interface, with identical Schmid factors of 0.31. These are the same slip planes observed in single-crystal Cu (SC-Cu) along the [111] direction. Therefore, although the presence of flat interfaces provides preferential slip nucleation sites near the interface, the overall deformation behavior in the Cu layer interior is not significantly modified. In comparison, the deformation behavior of Cu/Ta multilayers with KS112 interfaces is very different from the observed flat interfaces. As shown in Figure 4(b), for KS112 interfaces primary P1-($\overline{1}$11) and P1-(1$\overline{1}$1) slip planes, with a maximum Schmid factor of 0.39, are activated in the Cu layer interior. The activation of secondary S1-(11$\overline{1}$) slip plane, with a smaller Schmid factor of 0.31, is also observed, and mostly observed near the Cu/Ta interface. Zhang *et al*. also observed the activation of such secondary slip planes in Cu/Nb multilayers with the KS112 interface under shock compression, and argued that such dissociation is more favorable than the nucleation of new Shockley partials from the interface, despite its lower Schmid factor^44^. In addition, the pre-existing stacking faults are observed to extend from the interface to the Cu layer interior, triggering another secondary S2-(111) slip plane with a Schmid factor of 0. As shown in Figure 4(c), such extension of pre-existing stacking faults is also observed for the OT interfaces, along with two primary slip planes: P1-($\overline{1}$11) and P1-(1$\overline{1}$1). Table 2 lists the atomic fraction of atoms belonging to different slip planes, where the transition of the dominant slip planes from primary ones to secondary ones can clearly be seen with the decrease of interface spacing. These slip planes are listed in Supplementary Note 3. Therefore, the presence of faceted interfaces is observed to lead to the activation of a rich combination of primary and secondary slip planes, along with the extension/growth of pre-existing dislocations at the interface.

**Table 2:** Atomic fraction of atoms belonging to different slip planes at the end of SII (~20 ps) in Cu/Ta multilayers with KS112-case1. The number are shown for different interface spacing (λ).

λ (nm)	P1, m	P2, m	S1, m	S2, m
47	28.7%	32.9%	36.5%	1.9%
23	8.6%	45.2%	37.0%	9.2%
16	5.4%	40.2%	44.1%	10.2%
6	1.4%	5.4%	76.0%	17.3%

**Figure 5:**
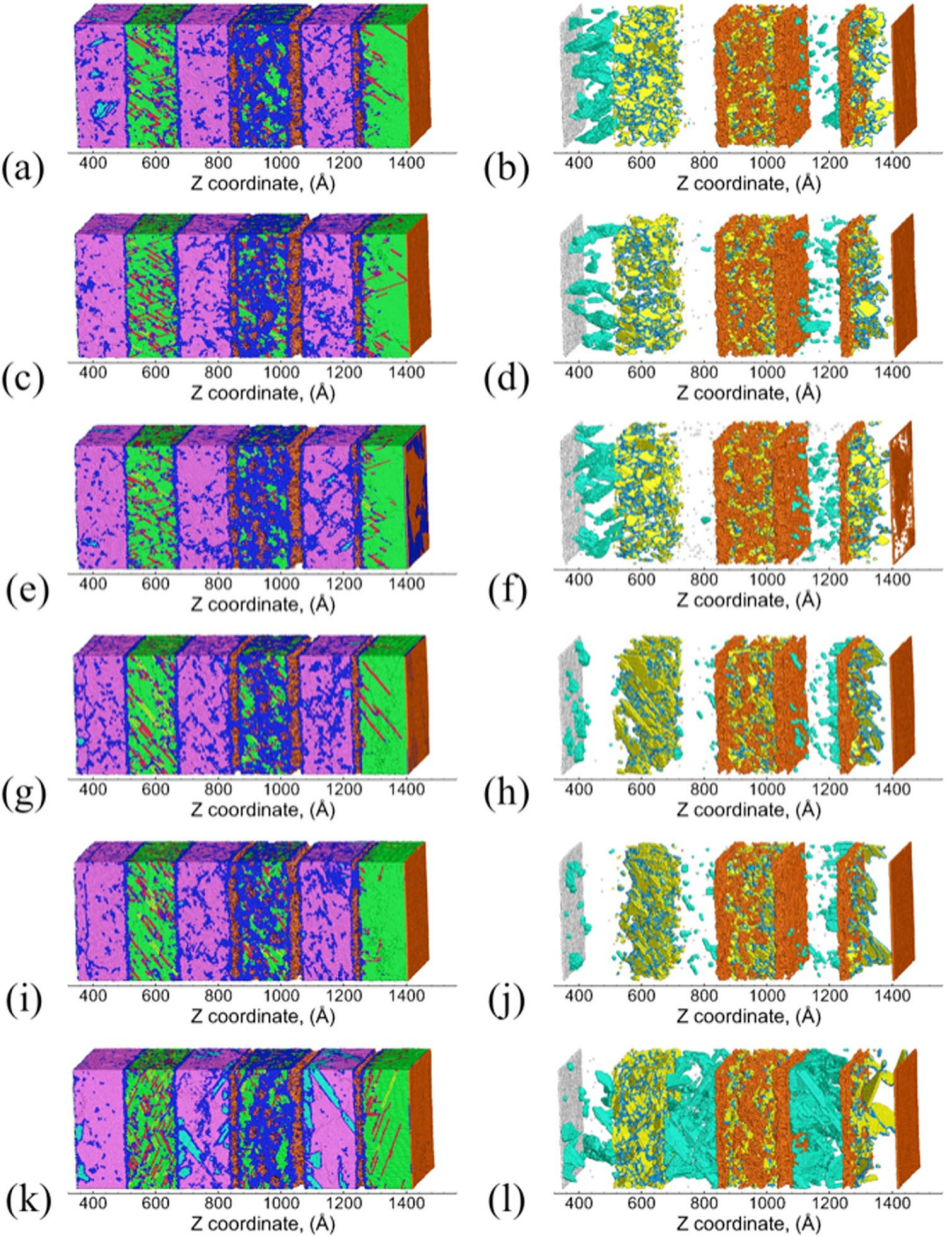
Snapshots of Cu/Ta multilayers at an interface spacing of 16 nm at the time of spall failure (~30 ps): (**a**,**b**) KS, (**c**,**d**) NW, (**e**,**f**) KS_2_, (**g**,**h**) KS112-case1, (**i**,**j**) KS112-case2, (**k**,**l**) OT. Right-hand panels show the distribution of defects (Cu twin faults, Cu twinning partials, and Ta twin faults) and damage (Cu surfaces and Ta surfaces). Atoms are colored as in Figure 3.

During SIII, the refracted wave propagates back, interacts with the tail of the release wave, and generates a tensile pressure zone. As the tensile pressure accumulates, voids start to nucleate in local regions with high-stress concentrations. These voids continue to grow and coalesce in SIV, and lead to the complete failure of the microstructure. Figure 5 shows the snapshots of the Cu/Ta multilayers and the corresponding defect microstructures at the time of spall failure (~30 ps). Due to the stress relaxation in the microstructure, most stacking/twin faults in the Cu layers and twin faults in the Ta layers nucleated during SI and SII (Figure 3) have already been annihilated. For all the Cu/Ta multilayers, spall failure occurs at the interfaces as well as in the interior of the 2nd Cu layer. The damage (voids) volume fraction distribution across the Z length of the sample is shown in Supplementary Note 4. The voids in the Cu layer interior result from the multiple slip systems that intersect one another, whereas the voids at the Cu/Ta interfaces result from the lower resistance of interface to void nucleation. However, the Ta layers are mostly void-free, suggesting that Ta layers are stronger, and more resistant to void nucleation. Table 3 lists the peak compressive pressure experienced by the Cu layers, and the maximum tensile pressure in the Cu layers that is defined as the spall strength. The values are also listed for SC-Cu and SC-Ta for comparison. The spall strength of SC-Ta is ~20 GPa, which is two times that of SC-Cu. As a result, during spallation voids are nucleated at the Cu/Ta interfaces and the Cu layer interior first, and their growth leads to stress relaxation, preventing the multilayered microstructure from attaining a tensile stress high enough to nucleate voids in the Ta layers. Therefore, despite the differences in the interface type/structure and the associated deformation behavior, the resulting damage (voids) distribution and failure behavior are very similar for all Cu/Ta multilayers. The spall strengths for the flat interfaces are found to be ~0.6 GPa higher than that of SC-Cu along the [111] direction. Under the same impact velocity, this increased spall strengths of Cu/Ta multilayers arise from the increased shock pressure, namely, higher compressive pressure achieved (~70 GPa) than that of SC-Cu (~45 GPa). As compared to the flat interfaces, the spall strengths for the faceted interfaces are slightly higher. However, the increase in spall strengths over SC-Cu in the corresponding direction ([112] for KS112 interfaces, and [001] for the OT interface) is only ~0.2 GPa. Therefore Cu/Ta multilayers can achieve higher spall strengths than SC-Cu, and such strengthening effects are more significant for the flat interfaces as compared to the faceted interfaces.

**Table 3:** Density of Stair-rod partial and twinning partial (10^17^/m^2^) at the spall plane, as well as peak compressive pressure (P_max_) and spall strength in the Cu layers (σ_spall_, GPa) for the Cu/Ta multilayers at an interface spacing of 16 nm. For comparison, the values are also listed for SC-Cu along [111], [112] and [110] direction.

Interface	Stair-rod	Twin	P_max_	σ_spall_
KS	0.16	0.29	70.69	10.73
NW	0.18	0.31	70.59	10.71
KS_2_	0.17	0.27	70.30	10.86
KS112-case1	0.18	0.62	71.43	11.01
KS112-case2	0.17	0.54	71.82	11.02
OT	0.03	0.20	69.55	10.94
SC-Cu [111]	0.12	0.27	44.70	10.06
SC-Cu [112]	0.32	1.30	55.92	10.76
SC-Cu [110]	0.05	0.57	63.90	10.74

**Figure 6:**
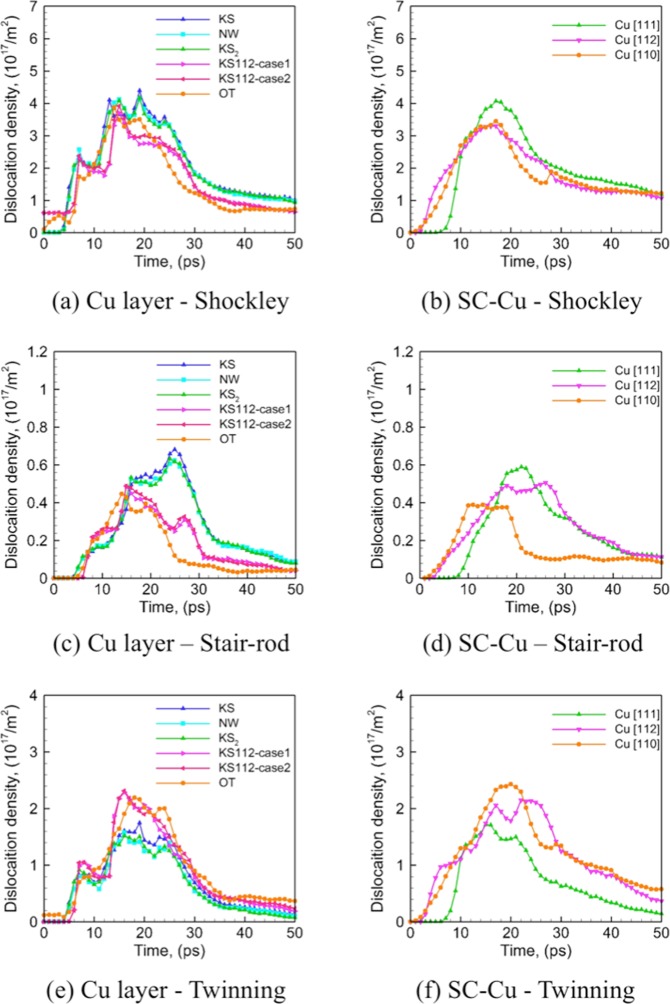
Evolution of overall density of dislocations (Shockley partials, Stair-rod partials, twinning partials) in the Cu layers for Cu/Ta multilayers at an interface spacing of 16 nm (left-hand panels) as compared to SC-Cu (right-hand panels): (**a**) Cu layer – Shockley partial, (**b**) SC-Cu – Shockley partial, (**c**) Cu layer - Stair-rod partial, (**d**) SC-Cu - Stair-rod partial, (**e**) Cu layer - twinning partial, (**f**) SC-Cu - twinning partial.

**Figure 7:**
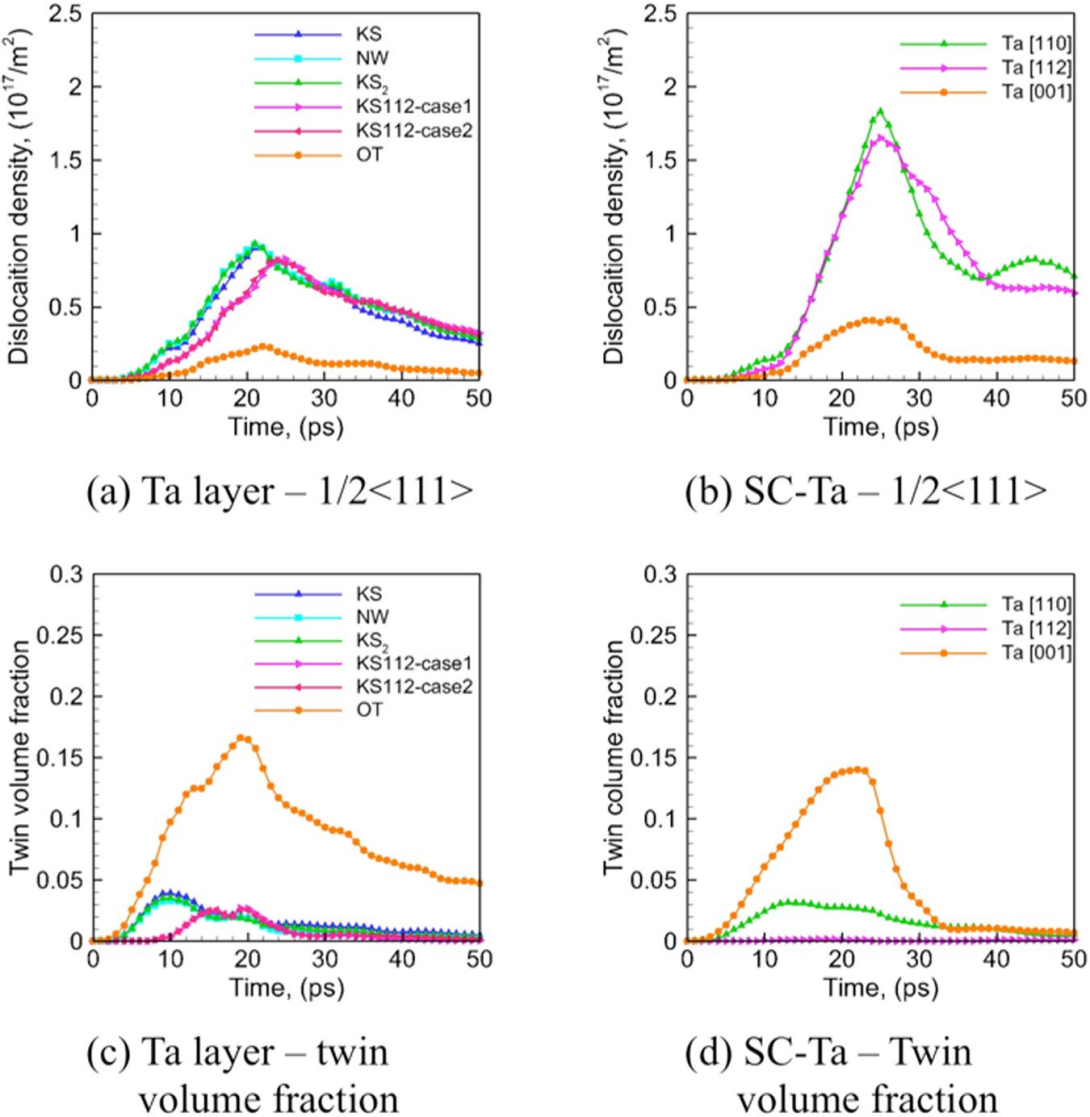
Evolution of overall density of dislocations (with Burgers’ vector of 1/2 〈111〉) and twin volume fraction in the Ta layers for Cu/Ta multilayers at an interface spacing of 16 nm (left-hand panels) as compared to SC-Ta (right-hand panels): (**a**) Ta layer – 1/2 〈111〉, (**b**) SC-Ta – 1/2 〈111〉, (**c**) Ta layer – twin volume fraction, (**d**) SC-Ta – twin volume fraction.

The evolution of dislocation densities in the Cu layers (Shockley partials, Stair-rod partials, and twinning partials) is characterized and plotted in Figure 6. The densities of these dislocations are observed to increase rapidly as the shockwave propagates through the Cu layers during SI and SII (up to 20 ps), and then decrease when it reaches the Cu/Ta interfaces that partially absorb the incoming dislocations. The dislocation densities reach peak values towards the end of SII as the shockwave reaches the rear surface, and then starts to decrease during SIII and SIV. Despite the differences in the initial dislocation densities, the densities of Shockley partials and twinning partials are similar for all the interfaces. In contrast, the Stair-rod partial densities are much lower for the faceted interfaces as compared to the flat ones, which may contribute to the higher spall strengths for the faceted interfaces, since Stair-rod partials are believed to provide nucleation sites for voids during spallation^54^. A comparison of the densities of Stair-rod partials and twinning partials at the spall plane (the region where voids nucleate) at the time of spall failure (~30 ps) is shown in Table 3. When compared to SC-Cu in the corresponding direction, the flat interfaces render similar densities of twinning partials at the spall plane, whereas the faceted ones render much lower values, which likely explains the less significant strengthening effects of the faceted interfaces as observed. Figure 7 shows the evolution of dislocation densities with Burgers’ vector of 1/2 〈111〉 and twin volume fraction in the Ta layers, as compared to the SC-Ta. It can be seen from Figures. 7(a),(c) that, the OT interface shows a much lower dislocation density and much higher twin volume fraction than all the other interfaces. This suggests that in Cu/Ta multilayers, the deformation behavior is twinning dominated for the OT interface and dislocation slip dominated for all other interfaces. Figure 7(b),(d) indicate that this trend is in line with SC-Ta: [001] direction that corresponds to the OT interface shows deformation twinning dominated behavior, whereas [110] and [112] direction that corresponds to the other interfaces show dislocation slip dominated behavior. However, the variation of dislocation density, as well as twin volume fraction in the Ta layers is very different from SC-Ta. A significant decrease in the dislocation density is observed for Cu/Ta multilayers (Figure 7(a),(b)), suggesting that dislocation nucleation is suppressed in the Ta layers. Twin volume fraction for the flat interfaces is similar to that observed in SC-Ta along [110] direction, whereas for the faceted interfaces the twin volume fraction is higher than that observed in SC-Ta along [112] and [001] direction (Figure 7(c),(d)). Moreover, in SC-Ta along [001] direction, most of the twins nucleated in SI and SII are annihilated during SIII and SIV, due to the relaxation of the shear stress. Such de-twinning has been reported for Ta^23^ and is believed to be responsible for the lack of twinning observed in shock-recovered microstructures in previous investigations^55^. However, this is not the case for the Cu/Ta multilayers with the OT interface, where most of the twins are retained. This suggests that the OT interface can significantly suppress the de-twinning in the Ta layers. The twin volume fraction in the Ta layers at the time of spall failure (~30 ps) is found to be much higher for the multilayer with OT interface (~0.10) as compared to SC-Ta along [001] direction (~0.01), as detailed in Supplementary Note 5. These results suggest that in Cu/Ta multilayers, Cu/Ta interfaces, especially that of faceted ones, significantly affects the overall deformation behavior in both the Cu and the Ta layers.

### Effects of interface spacing

**Figure 8:**
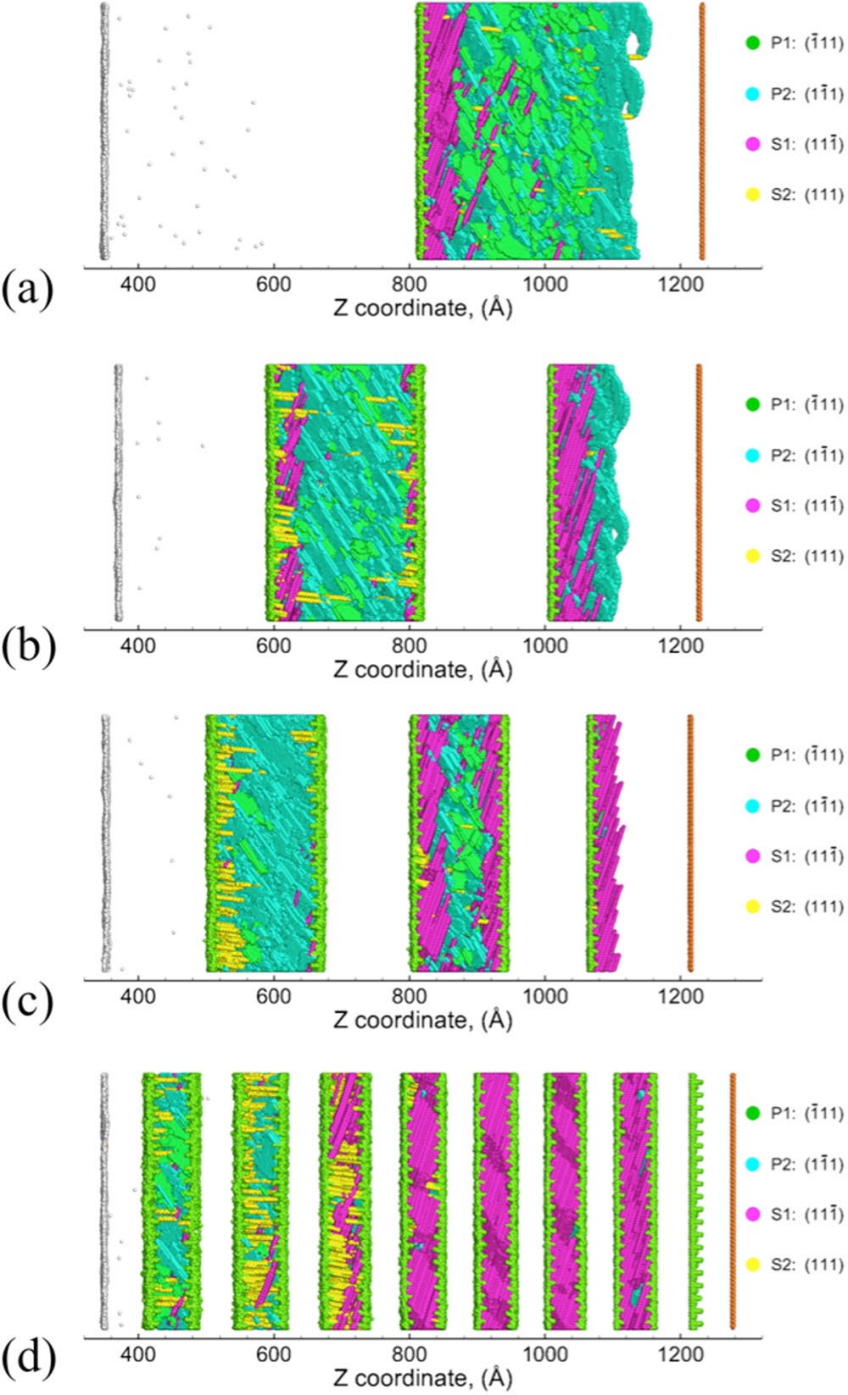
Snapshots showing the activated slip planes in Cu/Ta multilayers with KS112-case1 interface at the end of SII (~20 ps): (**a**) 47 nm, (**b**) 23 nm, (**c**) 16 nm, (**d**) 6 nm. Only atoms corresponding to the slip planes in the Cu layers (Cu stacking/twin faults) and damage (Cu surfaces and Ta surfaces) are shown. In the Cu layers atoms are colored based on the slip planes they correspond to, as shown on the right. Atoms corresponding to the Cu/Ta interfaces are shown as light green.

The effects of interface spacing on the deformation and spall behavior are investigated by considering Cu/Ta multilayers with a range of interface spacings: of 47 nm, 23 nm, 16 nm, and 6 nm is discussed here. Figure 8 shows the snapshots of the Cu/Ta multilayers and the corresponding defect microstructures at the end of SII (20 ps), using the KS112-case1 interface as an example. At interface spacings greater than 6 nm (Figure 8(a)–(c)), the activated slip planes in the Cu layer interior comprise mostly of the primary P1-($\overline{1}$11) and P2-(1$\overline{1}$1) slip planes, whereas the activated slip planes near the Cu/Ta interfaces comprise mostly of secondary S1-(11$\overline{1}$) and S2-(111) slip planes. Notably, when the interface spacing decreases to 6 nm (Figure 8(d)), the activated slip planes are mostly secondary S1-(11$\overline{1}$) and S2-(111) planes near the Cu/Ta interfaces. At such small interface spacing, the stacking/twin faults nucleated from the Cu/Ta interfaces propagate across the entire Cu layer, thus suppressing homogeneous dislocation nucleation and propagation in the Cu layer interior. It is also noted from Figure 8(d) that the stacking/twin faults nucleated from both Cu/Ta interfaces enclosing each Cu layer are mostly aligned parallel to each other along S1-(11$\overline{1}$), showing an “alternate emission” pattern^15^. Such alignment arises from and interaction of the stress field of two Cu/Ta interfaces at such small interface spacing^56^. These results suggest a transition from homogeneous mode to interface-dominated heterogeneous deformation mode. This transition is primarily driven by the variation in interface spacing and is observed to occur for all Cu/Ta multilayers, regardless of the interface type.

**Figure 9:**
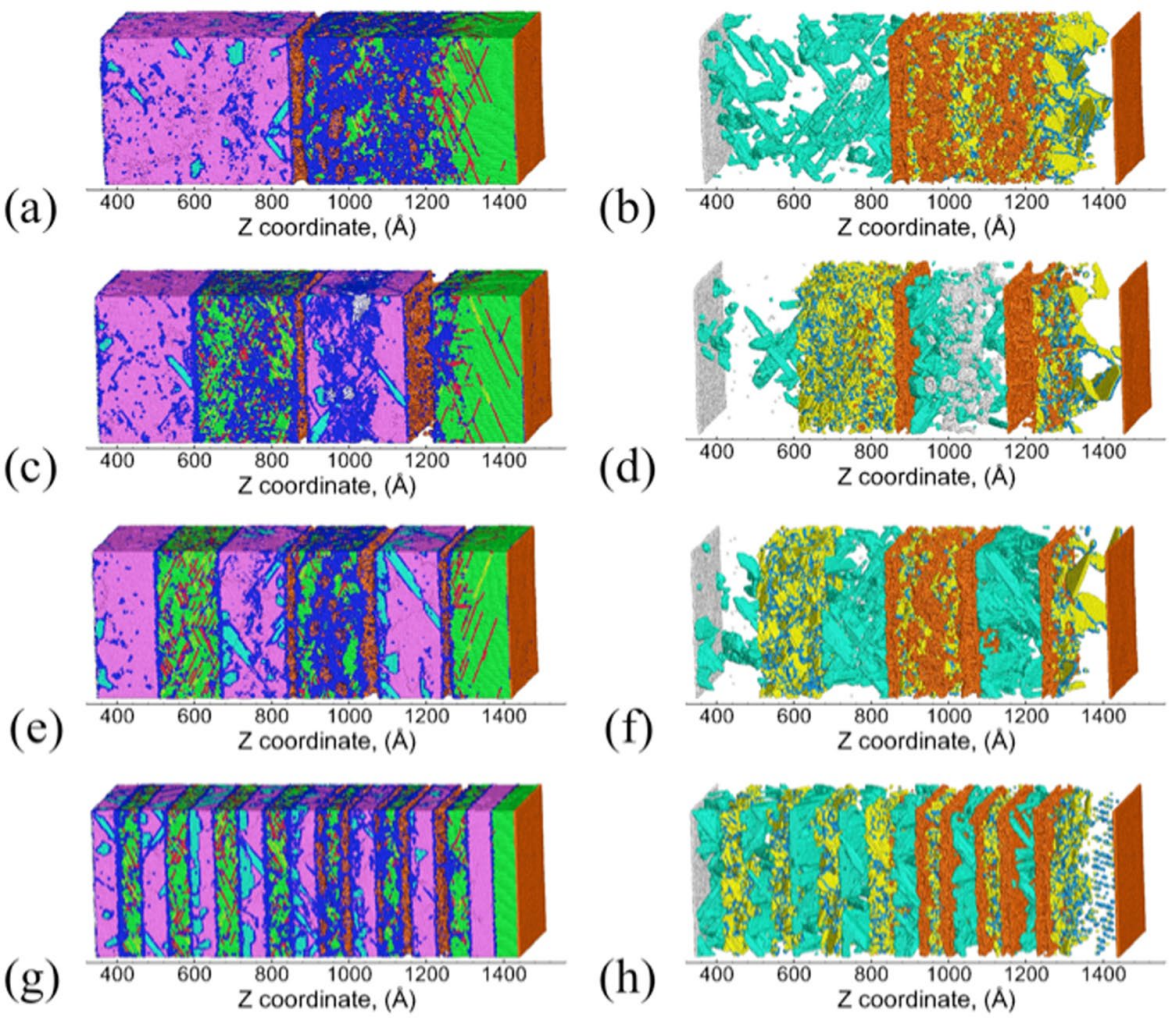
Snapshots of Cu/Ta multilayers with OT interface at the time of spall failure (~30 ps): (**a**,**b**) 47 nm, (**c**,**d**) 23 nm, (**e**,**f**) 16 nm, (**g**,**h**) 6 nm. Right-hand panels show the distribution of defects (Cu twin faults, Cu twinning partials, and Ta twin faults) and damage (Cu surfaces and Ta surfaces). Atoms are colored as in Figure 3.

**Figure 10:**
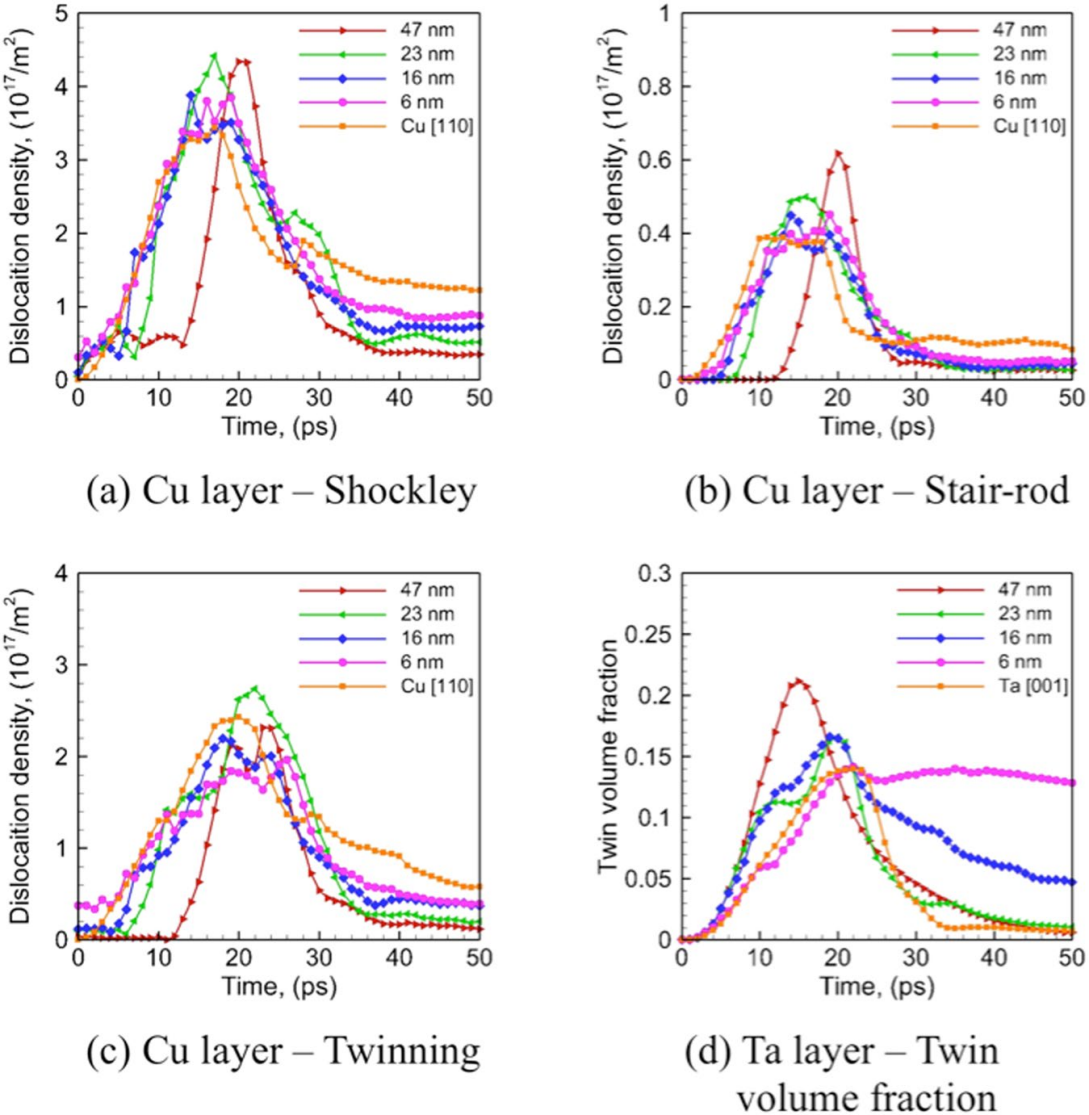
Evolution of overall density of dislocations in the Cu layers and twin volume fraction in the Ta layers for Cu/Ta multilayers with OT interface: (**a**) Cu layer - Shockley, (**b**) Cu layer - Stair-rod, (**c**) Cu layer - twinning partial, (**d**) Ta layer – twin volume fraction.

The effects of interface spacing on void nucleation and spallation behavior of the Cu/Ta multilayers are further examined, using the OT interface as an example. Figure 9 shows the snapshots of the Cu/Ta multilayers with the OT interface, and Figure 10 shows the evolution of dislocation densities in the Cu layers and twin volume fraction in the Ta layers. From Figure 9 it can be observed that at interface spacings greater than 6 nm, voids are located at the Cu/Ta interfaces as well as in the Cu layer interior, as has been discussed for an interface spacing of 16 nm. However, as the interface spacing decreases to 6 nm, voids are mostly located at the Cu/Ta interface. More detailed demonstration of the damage (voids) volume fraction distribution is shown in Supplementary Note 7. The dislocation densities in the Cu layers do not vary significantly with interface spacing. Although the volume fraction of twins nucleated in SI and SII is similar to SC-Ta along [001] direction and does not vary much with interface spacing, the annihilation of these twins is largely suppressed at smaller interface spacing. This is especially true as the interface spacing decreases to 6 nm, where the twin volume fraction stays nearly constant during SIII and SIV. Therefore, for Cu/Ta multilayers with the OT interfaces, as the dislocation densities in the Cu layers vary little with interface spacing, the substantially higher twin volume fraction in the Ta layers may contribute to the lower spall strengths observed at an interface spacing of 6 nm. Interestingly, at an interface spacing of 23 nm, spallation is observed to occur in the Ta layer interior as well (Figure 9(c),(d)). This is quite different from the other interfaces where spallation occurs only at the Cu side of Cu/Ta interface and the Cu layer interior, as discussed in Supplementary Note 6 and 8. The spallation in Ta layer observed for the OT interface is due to the significant twinning in the Ta layers, which provides preferential void nucleation sites. Moreover, the spall strength achieved in the Ta layer is 19.21 GPa in this case, which is much lower than the value of 22.75 GPa observed for SC-Ta along [001] direction. This is likely due to the profuse amount of twinning in the Ta layers, which provides preferential void nucleation sites and thus lowers the spall strength, as revealed for SC-Ta^57^.

These findings suggest that interface spacing plays a crucial role in dislocation nucleation and propagation, as well as void nucleation and spall behavior of the Cu/Ta multilayers. In particular, void nucleation and spall failure are observed to occur not only at the Cu/Ta interfaces, but also in the weaker Cu layer interior, or even in the much stronger Ta layer interior, depending on the interface spacing and structure. This behavior contradicts the general belief of preferential void nucleation at the bi-metallic interfaces for multilayered microstructures under shock loading conditions^45,58,59^. In addition, for the range of interface spacing considered, similar deformation and spall behavior are observed among all the flat interfaces, as well as among both the KS112 interfaces that share the same loading orientation but different local interface structure. This suggests that, under the same loading orientation, minor variations in the local interface structure do not significantly affect the overall deformation and failure behavior of the Cu/Ta multilayers.

### Overall trends in spall strength

**Figure 11:**
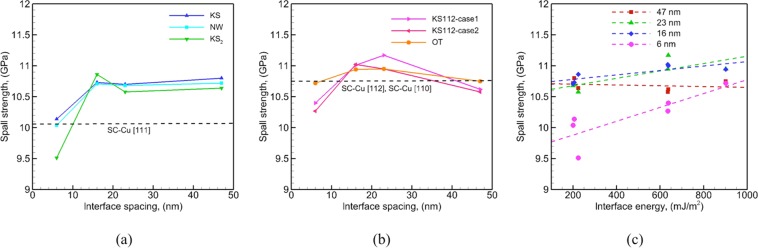
Variation of the spall strength with interface spacing: (**a**) flat interfaces, and (**b**) faceted interfaces. The values of SC-Cu along [111], [112], and [110] direction are also marked in the plots for comparison. As the spall strengths along [112] and [110] direction are very close (10.76 GPa, and 10.74 GPa, respectively), the average value is shown in (**b**) as the spall strength of [112] and [110] direction, and (**c**) variation of the spall strength with interface energy.

The variation of spall strengths of the Cu/Ta multilayers with interface spacing is plotted in Figure11, with the results for flat interfaces and faceted interfaces plotted in (a) and (b), respectively. As shown in Figure 11(a), the spall strengths of Cu/Ta multilayers with flat interfaces with spacings greater than 6 nm are higher than SC-Cu and vary little with interface spacing. Such strengthening effects likely arise from the higher shock pressure experienced by the multilayered microstructure as compared to SC-Cu under the same impact velocity. Moreover, the shock pressures are similar for all interface spacings, and so are the resulting spall strengths. As interface spacing decreases to 6 nm, the spall strengths of the Cu/Ta multilayers are similar to or lower than that of SC-Cu due to the interface-dominated failure mode and the weak nature of the Cu/Ta interfaces. In comparison, the faceted interfaces show a different variation. As shown in Figure 11(b), the spall strength values of Cu/Ta multilayers with faceted interfaces are similar or lower than that of SC-Cu at interface spacings of 47 nm and 6 nm, and higher at intermediate interface spacings of 23 nm and 16 nm. Therefore, similar strengthening and weakening effects can be observed for faceted interfaces as well, except the unexpected weakening effects observed at an interface spacing of 47 nm. Also, the strengthening is less significant (~0.2 GPa) for the faceted interfaces as compared to the flat ones (~0.6 GPa), as the strengthening effects for the flat interfaces also arise from the higher twinning partial density in the Cu layers. For the given systems and loading conditions studied here, the optimal interface spacing, i.e. the spacing that renders the highest spall strength values, is located at either 16 nm or 23 nm for nearly all the interfaces.

It is also of interest to investigate how static properties such as interface energy affect the spall strengths of the Cu/Ta multilayered microstructures. Figure 11(c) shows the variation of spall strengths plotted against interface energy at various interface spacings. The plot shows that, at an interface spacing of 6 nm, high-energy faceted interfaces show higher spall strengths as compared to low-energy flat interfaces. As discussed before, the deformation mechanism is interface-dominated at this small interface spacing. Therefore, the variation in atomic structure and energy of the interface, for example, interface energy, is likely to contribute to the variation in spall strength, as reported previously^60^. However, at interface spacings greater than 6 nm, the spall strengths of all the multilayered microstructures are very similar. This insensitivity of spall strength to interface spacings exceeding 6 nm is attributed to the bulk-dominated spall failure. However, more data is needed to identify any correlations between spall strength, dislocation density, and interface energy of these multilayered microstructures.

## Conclusions

The role of interface type and spacing on the wave propagation, dislocation nucleation and propagation, and void nucleation and spall behavior of the Cu/Ta multilayered microstructures is investigated with MD simulations. The major findings of this work include:

1. Under shock loading condition, flat interfaces show similar deformation behavior as SC-Cu, whereas faceted interfaces result in the activation of multiple secondary slip systems with low Schmid factor.
2. The overall deformation mechanism transitions from homogeneous dislocation nucleation to interface-assisted heterogeneous dislocation nucleation as the interface spacing is decreased to 6 nm. Accordingly, the resulting spall behavior transitions from bulk failure (in the Cu layer interior) to interface failure (at the Cu/Ta interface). Failure could even in the stronger Ta layer in the case of OT interface, due to the profuse twinning in the Ta layer.
3. The Cu/Ta interfaces affect the twinning propensity in the Cu and Ta layers differently. Flat interfaces enhance twinning in the Cu layers, and do not significantly affect twinning in the Ta layers. Faceted interfaces restrict twinning in the Cu layers, yet suppress de-twinning in the Ta layers, resulting in significant twinning in the multilayered microstructure at the time of failure.
4. As compared to faceted interfaces, flat interfaces result in more significant increase in spall strengths as compared to SC-Cu. Under the same loading orientation, although interface structure does not alter the overall deformation and failure behavior, it does affect the spall strengths of the Cu/Ta multilayered microstructures at an interface spacing of 6 nm, when failure is restricted at the interface.
5. The spall strength values vary with interface spacing for given system size and loading conditions wherein peak values are observed at a spacing of 16–23 nm, and lowest values are observed at spacings of 6 nm and lower.

## Methods

The interface energy is calculated as:

1 $${\rm{\gamma }}=\frac{{E}_{slab}-{N}_{Cu}{E}_{Cu}-{N}_{Ta}{E}_{Ta}}{A}$$

where *E*_*slab*_ is the total energy of the slab containing the given Cu/Ta interface, *N*_*cu*_ and *E*_*Cu*_ are the number and cohesive energy of FCC Cu atoms, and *N*_*Ta*_ and *E*_*Ta*_ are the number and cohesive energy of BCC Ta atoms, and A is the interface area.

The MD simulations are carried out with the open source software LAMMPS^61^. The atomic interaction is described by the angular-dependent interatomic potential^62^. This potential provides an accurate description of the structural stability and high strain-rate deformation behavior, including stacking fault energetics and shock Hugoniot^63^. Cu and Ta crystals are joined to construct the multilayered structure, based on the orientation relationship. The lateral dimensions are optimized to minimize the in-plane strains.

For the initial Cu/Ta multilayered microstructures, periodic boundary conditions are applied on X and Y direction, whereas Z direction is kept free. Prior to shock, the multilayered microstructures are first equilibrated at 300 K and zero pressure for 50 ps. The system is shocked by driving a 5 nm thick piston at the left end of sample (shown by grey atoms in Figure 2) inwards into the sample (to the right) at a fixed velocity of 1 km/s. The shock loading (inward drive of piston) is applied for 10 ps. Then the velocity fix is removed, and the system is allowed to evolve under NVE ensemble. The leftmost most layer is always kept as Ta layer for all systems, therefore the shock loading is always initiated from the Ta layer, and propagates to the successive Cu and Ta layers. Starting from the leftmost Ta layer, the successive Ta and Cu layers are labeled as the 1st Ta layer, 1st Cu layer, 2nd Ta layer, 2nd Cu layer, … etc. A time step of 2 fs is chosen for all simulations.

The “dislocation extraction algorithm” (DXA)^64,65^ and “crystal analysis tool” (CAT)^66^ are used to characterize defects (Perfect, Shockley, Stair-rod, twinning partial dislocations, stacking faults, twin faults, surface/voids, etc). It should be noted that the twinning partials in FCC metals and Shockley partials have the same Burgers vector ($\frac{a}{6}\langle 112\rangle$) and hence, cannot be distinguished from each other. The methodology to characterize twining partials can be found in^28,30,31,40,41,54^. In addition, twins are characterized in Ta based on Euler angles that represent the local orientation of each atom. The calculated Euler angles are compared to the initial (reference) Euler angles. Atoms with significantly changed Euler angles as compared to the initial (reference) values, and with similar Euler angles as compared to its neighbors are identified as ‘twinned’ atoms. In identifying ‘twinned’ atoms, only the atoms inside the twinned region are considered, whereas atoms at twin boundaries are not included. The twin volume fraction is calculated as the total volume fraction of the ‘twinned’ atoms. More details regarding twinning characterization can be found in^32^. To remove the thermal noise, all snapshots are quenched to 0 K for visualization.

## Supplementary information

Supplemental Information.

## Data Availability

The datasets generated during and/or analyzed during the current study are available from the corresponding author on reasonable request.
