# Supplementary material for: Unraveling the Role of Interfaces on the Spall Failure of Cu/Ta Multilayered Systems
Source: Sci Rep. 2020 Jan 14;10:208. doi: 10.1038/s41598-019-57048-9 (PMC6959279; doi:10.1038/s41598-019-57048-9)
Supplement: Supplementary file 1 — Supplemental Information. [file 41598_2019_57048_MOESM1_ESM.pdf]

Unraveling the Role of Interfaces on the Spall Failure of Cu/Ta Multilayered Systems

Jie Chen [a], Suveen N. Mathaudhu [b], Naresh Thadhani [c] and Avinash M. Dongare [a,*]

[a] Department of Materials Science and Engineering, and Institute of Materials Science, University of Connecticut, Storrs, CT 06269, USA

[b] Mechanical Engineering, and Materials Science and Engineering, University of California, Riverside, CA 92507, USA

[c] Department of Materials Science and Engineering, Georgia Institute of Technology, Atlanta, GA 30332, USA

[*] Corresponding author.
Email address: dongare@uconn.edu (A.M. Dongare).

Supplementary Note 1: pre-existing dislocation densities

Table S1: Pre-existing dislocation densities ($10^{17}/m^2$) per interface for the Cu/Ta multilayers.

Interface	Shockley	Stair-rod	Twin
KS	0.00	0.00	0.00
NW	0.00	0.00	0.00
KS_2	0.00	0.00	0.00
KS112-case1	0.12	0.00	0.00
KS112-case2	0.12	0.00	0.00
OT	0.02	0.00	0.03

Supplementary Note 2: shock loading and propagation

The shockwave compression and propagation behavior are discussed in SI and SII. SI corresponds to the loading of the compression wave in the system for a duration of 10 ps (shock pulse duration), whereas SII corresponds to the release of the compression wave starting at 10 ps and ends as the compression wave propagates towards the rear surface at a time of ~20 ps. Fig. S1 shows the evolution of normal pressure component (Z pressure profile) at intermediate times during SI and SII. The Z pressure profile are shown for times of 5 ps, 8 ps for SI, and 11 ps, 14 ps, 17 ps for SII. To show the variation in the Z pressure as the shockwave travels across multiple Cu/Ta interfaces, the pressure profiles are shown as solid lines for the portion of the multilayers belonging to the Ta layers, and dashed lines for the Cu layers. As shown in Fig. S1, since the shock loading is initiated from the Ta layer, the compressive Z pressure reaches ~95 GPa in the 1st Ta layer for all the Cu/Ta multilayers at 5 ps (orange lines). A clear two-wave (elastic and plastic waves) structures can be identified, with an elastic front leading the plastic wave. The transmitted Z pressure is significantly reduced as the shockwave propagates from the 1st Ta layer to the 1st Cu layer, due to the impedance mismatch of the Ta and Cu component layers (lower impedance of Cu as compared to Ta) [23, 44]. Subsequently, as the elastic front propagates from the Cu layers to the 2nd Ta layer at 8 ps (blue lines) and the 3rd Ta layer at 14 ps (purple lines), the resulting Z pressure is slightly increased. As a result, the pressure profiles show significant discontinuity between the solid lines representing Ta layers and dashed lines representing Cu layers, which is typical for multilayered microstructures [44]. The Z pressure of the elastic front in Cu layers continuously decreases as it propagates from the 1st Cu layer at 5 ps

(orange lines), to the 2nd Cu layer at 11 ps (green lines), and the 3rd Cu layer at 17 ps (pink lines), as indicated by the dashed black lines. At these times, substantial oscillation in the Z pressure profile at the elastic front can be observed, which could be attributed to the interaction of the elastic front with the Cu/Ta interfaces, similar to that observed in bi-crystal Cu with incoherent twin boundaries [45]. At 17 ps, right before the shockwave reaches the rear surface, the approximate locations of the elastic front, HEL and plastic front are marked by arrows in the figures. The resulting Z pressure of elastic front (HEL values) at 17 ps is ~40 GPa for all the flat interfaces (KS, NW, KS_2), ~25 GPa for both KS112 interfaces (KS112-case1, KS112-case2), and ~40 GPa for the OT interface. These values compare well with the calculated HEL values calculated from the rear surface profile. Therefore, the wave attenuation and elastic precursor decay is highly dependent on the interface type.

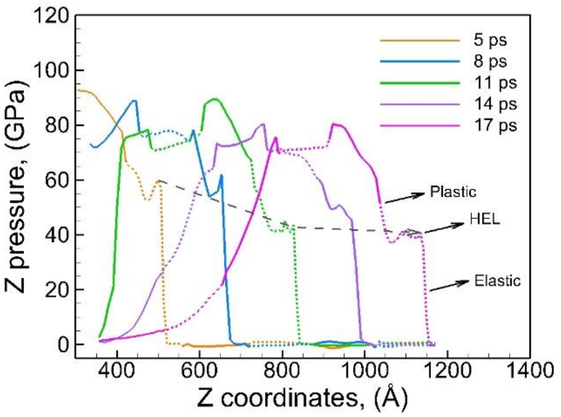

(a)

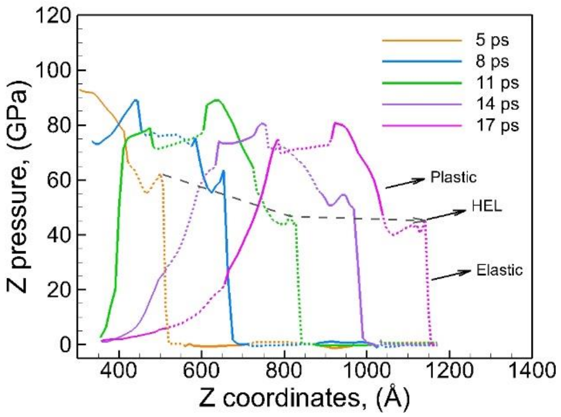

(b)

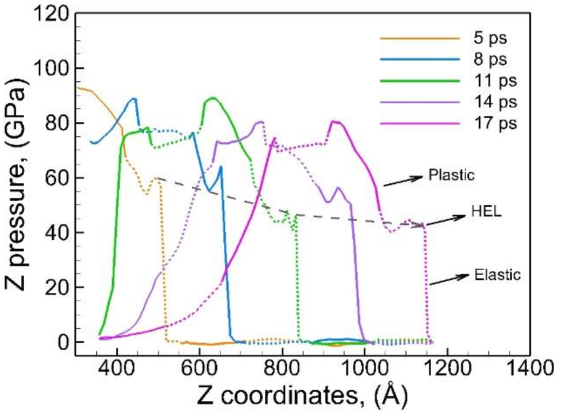

(c)

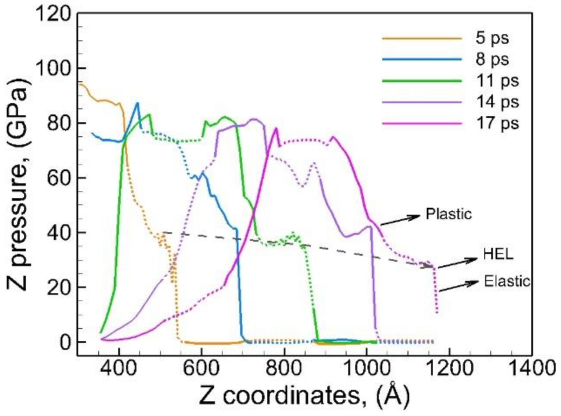

(d)

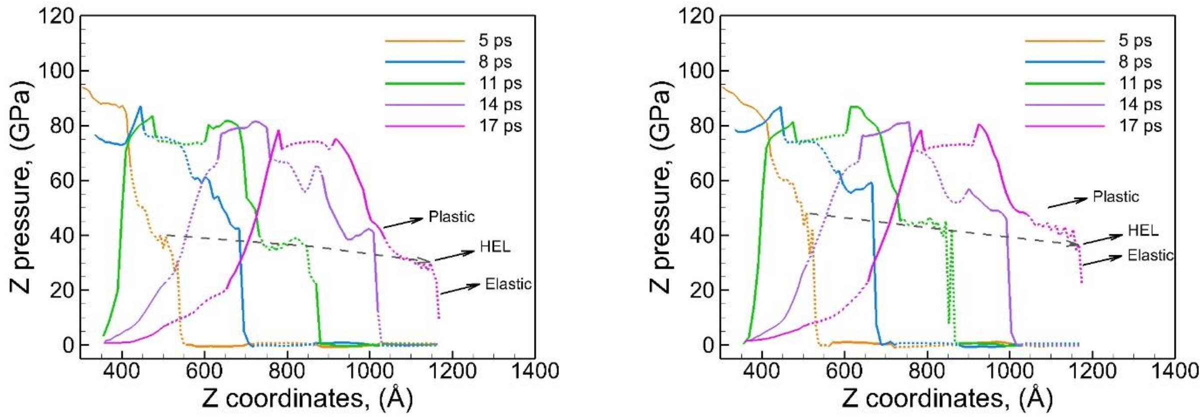

(e) (f)

Fig. S1. Shock wave profiles at intermediate times during SI and SII for Cu/Ta multilayers at an interface spacing of 16 nm: (a) KS, (b) NW, (c) KS_2, (d) KS112-case1, (e) KS112-case2, (f) OT. The pressure profiles are shown as solid lines for the portion of the multilayers belonging to the Ta layers, and dashed lines for the Cu layers, in order to show the variation in the Z pressure as the shockwave travels across multiple Cu/Ta interfaces. The dashed black lines show the decay of the elastic precursor as the elastic front propagates to the 1st Cu layer (~5 ps), 2nd Cu layer (~11 ps), and 3rd Cu layer (~17 ps), and the approximate locations of the elastic front, HEL and plastic front are marked by arrows at a time of 17 ps as the shockwave is about to reach the rear surface.

Supplementary Note 3: activated slip systems and the corresponding Schmid factors

Table S2: Activated slip planes and the corresponding Schmid factors in the Cu layers for the Cu/Ta multilayers at an interface spacing of 16 nm. Primary slip planes are labeled P1, P2, P3, etc, and secondary slip planes are labeled S1, S2, etc.

**Interface**	**P1, m**	**P2, m**	**P3, m**	**S1, m**	**S2, m**
KS	$(\bar{1}11)$, 0.31	$(1\bar{1}1)$, 0.31	$(11\bar{1})$, 0.31	(111), 0	N/A
NW	$(\bar{1}11)$, 0.31	$(1\bar{1}1)$, 0.31	$(11\bar{1})$, 0.31	(111), 0	N/A
KS_2	$(\bar{1}11)$, 0.31	$(1\bar{1}1)$, 0.31	$(11\bar{1})$, 0.31	(111), 0	N/A
KS112-case1	$(\bar{1}11)$, 0.39	$(1\bar{1}1)$, 0.39	N/A	$(11\bar{1})$, 0.31	(111), 0
KS112-case2	$(\bar{1}11)$, 0.39	$(1\bar{1}1)$, 0.39	N/A	$(11\bar{1})$, 0.31	(111), 0
OT	$(\bar{1}11)$, 0.47	$(1\bar{1}1)$, 0.47	N/A	$(11\bar{1})$, 0	(111), 0

Supplementary Note 4: damage (voids) distribution for Cu/Ta multilayers

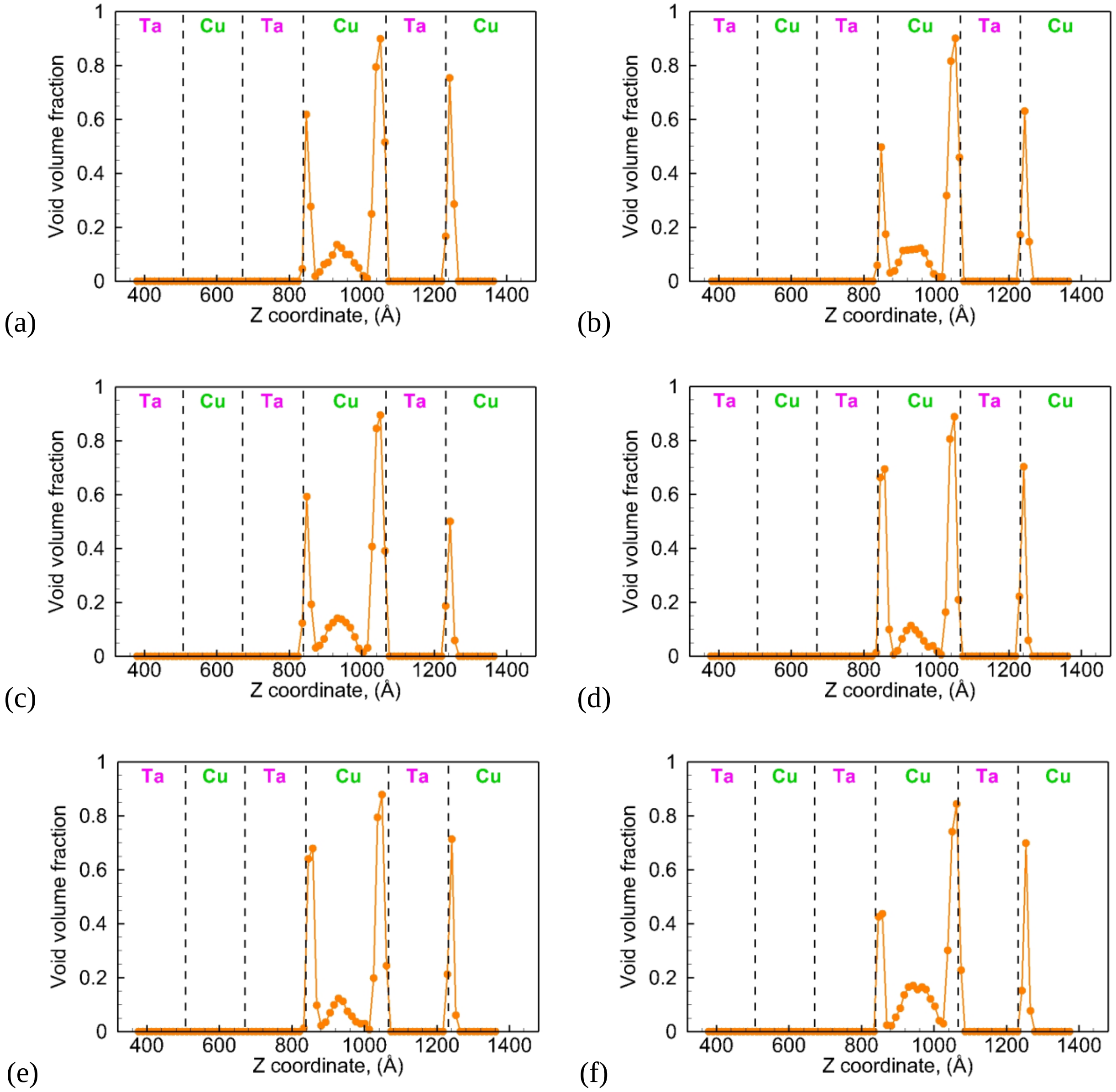

Fig. S2. Distribution of void volume fraction for Cu/Ta multilayers at an interface spacing of 16 nm at the time of spall failure (~30 ps): (a) KS, (b) NW, (c) KS_2, (d) KS112-case1, (e) KS112-case2, (f) OT. The Cu/Ta interfaces are marked by black dashed lines, and the Cu and Ta layers are marked out.

Supplementary Note 5: dislocation density and twin volume fraction for Cu/Ta multilayers

Table S3: Values of density of dislocations with Burgers vector 1/2 <111> ($10^{17}/m^2$) and twin volume fraction (f_{twin}) in the Ta layers for the Cu/Ta multilayers at an interface spacing of 16 nm. For comparison, the values are also listed for SC-Ta along [110], [112] and [001] direction.

Interface	1/2 <111>	f_{twin}
KS	1.29	0.013
NW	1.30	0.007
KS_2	1.29	0.009
KS112-case1	1.33	0.006
KS112-case2	1.23	0.005
OT	0.24	0.097
SC-Ta [110]	0.83	0.012
SC-Ta [112]	0.94	0.001
SC-Ta [001]	0.16	0.012

Supplementary Note 6: spall behavior and dislocation evolution for Cu/Ta multilayers with NW interface

To illustrate the effects of flat interfaces, Fig. S3 shows the snapshots of the Cu/Ta multilayers with NW interface and the corresponding defect microstructures at the time of spall failure (~30 ps). As shown in Fig. S3a – S3c, at interface spacings greater than 6 nm, due to the presence of both homogenous and heterogeneous dislocation activity, void nucleation is observed both at the Cu layer interior and the Cu/Ta interfaces. However, as interface spacing is decreased to 6 nm, as shown in Fig. S3d, the dominant deformation mode transitions to heterogeneous dislocation nucleation from the Cu/Ta interfaces, and void nucleation is restricted at the interface. Another noticeable change is the decrease in twin volume fraction in the Ta layers as the interface spacing is decreased. To reveal the above trends more clearly, Fig. S4 shows the evolution of the densities of Shockley partials, Stair-rod partials, and twinning partials in Cu layers, as well as twin volume fraction in the Ta layers for various interface spacing. As interface spacing is decreased, more local dips are observed in the plots, due to the presence of a greater number of Cu/Ta interfaces in the multilayered microstructure. The plots do not suggest a significant change in the overall density of these dislocations in the Cu layers with interface spacing, as shown in Fig. S3a – S3c. However, as can be seen in Table S4, at the time of spall

failure, the local densities of these dislocations at the spall plane show a substantial decrease with the decrease of interface spacing. At interface spacings greater than 6 nm, the densities of Shockley partials and twinning partials are higher than that of SC-Cu along [111] direction, resulting in higher spall strengths than SC-Cu. However, as the interface spacing is decreased to 6 nm, the densities of these dislocations decrease to a level similar to that of SC-Cu, resulting in spall strength values similar to SC-Cu. Moreover, as shown in Fig. S3d, the twin volume fraction in the Ta layers also decreases with the decrease in interface spacing, suggesting that the presence of NW interfaces reduces the twinning propensity in the Ta layers. However, due to the relatively small volume fraction, especially towards the end of SIII during spallation, twins in the Ta layers are not expected to have a significant impact on the spall behavior and the resulting spall strengths of Cu/Ta multilayers. The above trend observed for NW interface is representative of all the flat interfaces. Therefore, for Cu/Ta multilayers with flat interfaces, smaller interface spacing reduces the capability of the Cu/Ta multilayers to nucleate Shockley partials and twinning partials and results in interface-dominated failure mode, thus rendering lowered spall strengths.

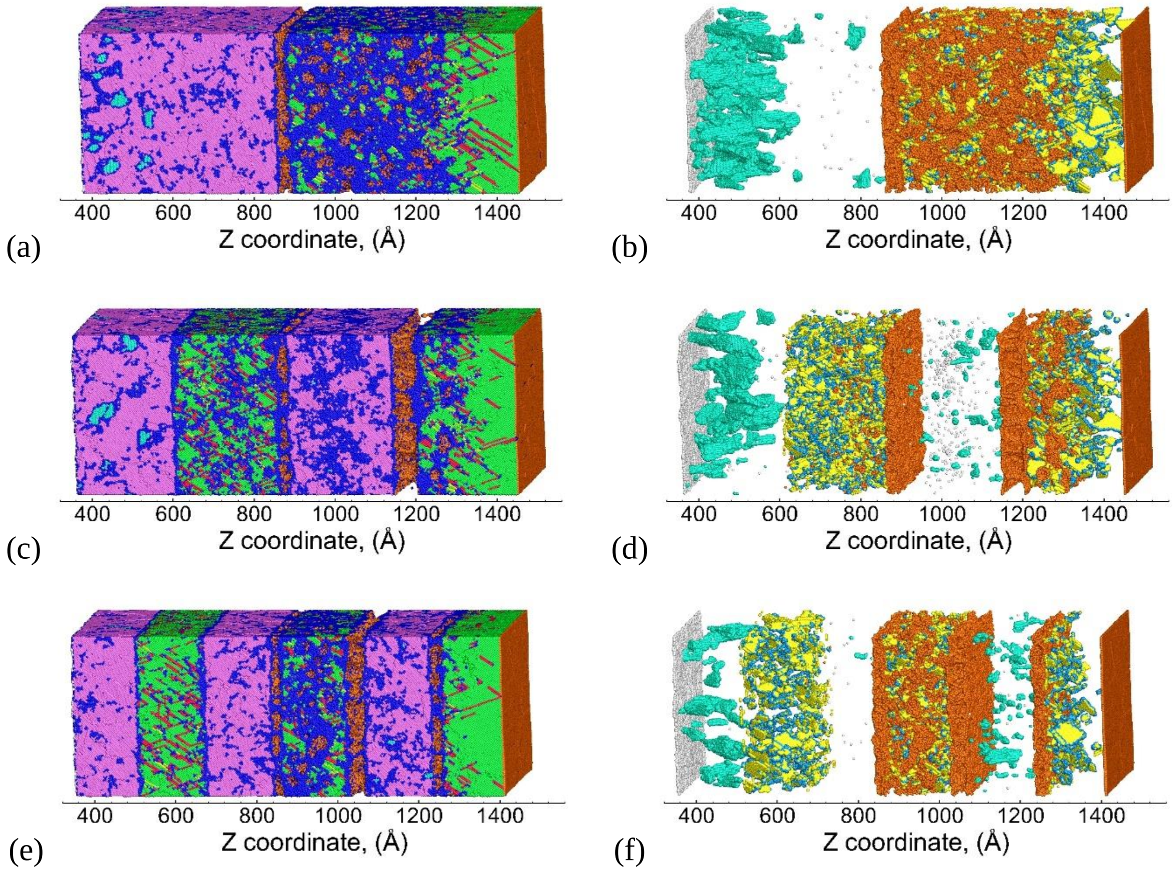

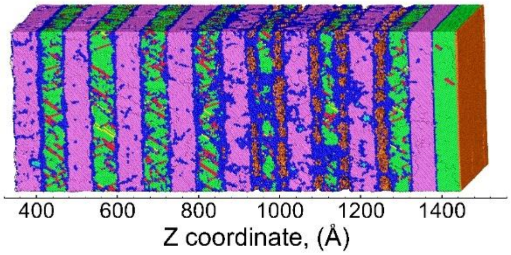

(g)

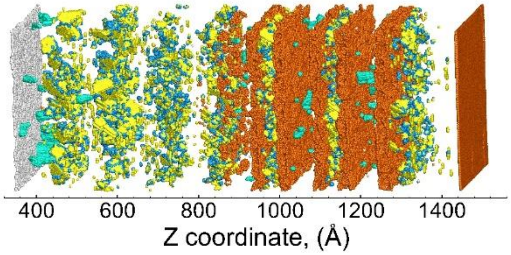

(h)

Fig. S3. Cu/Ta multilayers with NW interface at the time of spall failure (~30 ps): (a) – (b) 47 nm, (c) – (d) 23 nm, (e) – (f) 16 nm, (g) – (h) 6 nm. Right-hand panels show the distribution of defects (Cu twin faults, Cu twinning partials, and Ta twin faults) and damage (Cu surfaces and Ta surfaces). Atoms are colored as in Fig. 3.

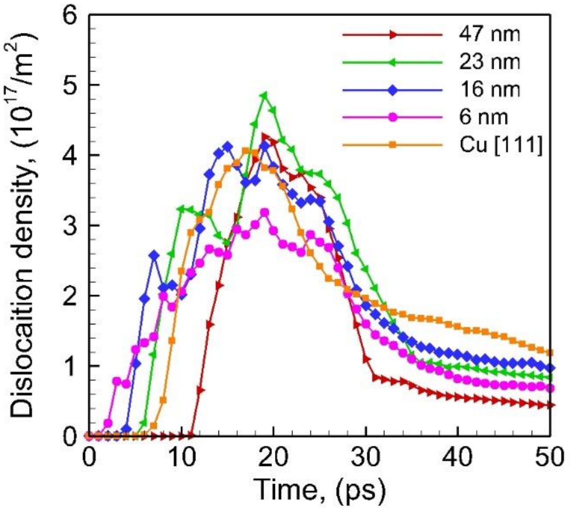

(a) Cu layer – Shockley partial

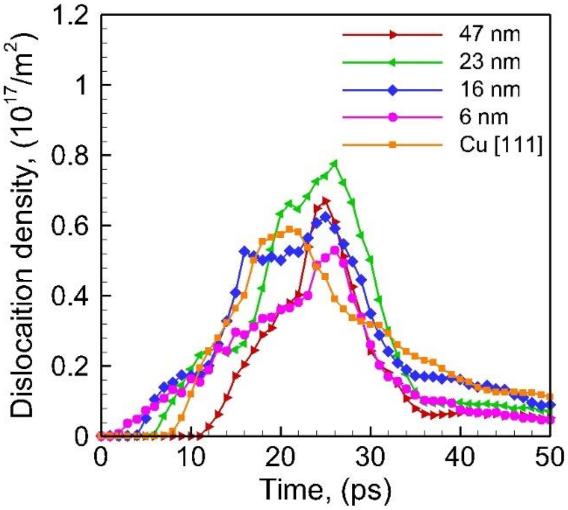

(b) Cu layer – Stairrod partial

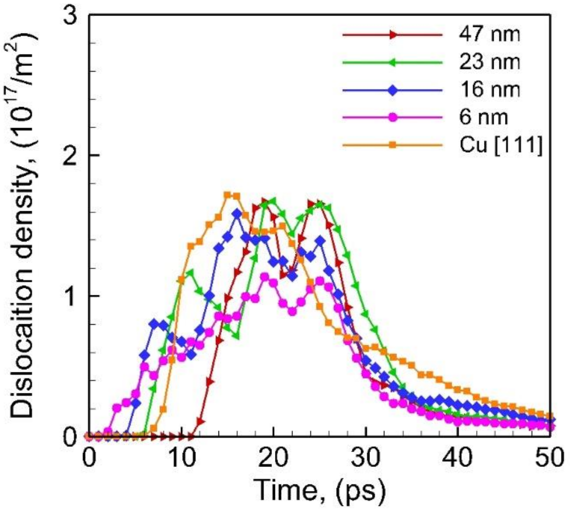

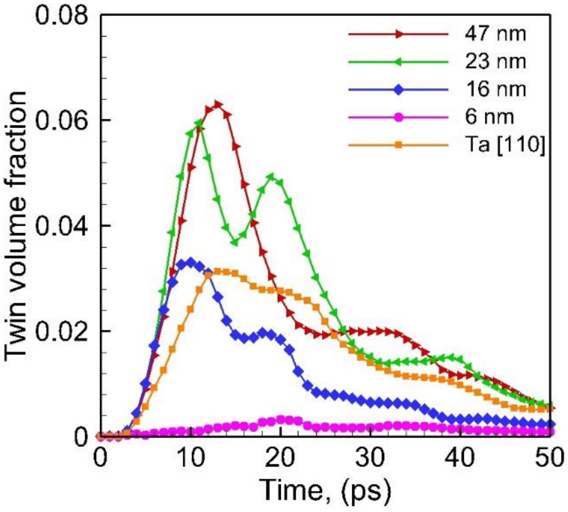

(c) Cu layer – twinning partial (d) Ta layer – twin volume fraction

Fig. S4. Evolution of overall density of dislocations in the Cu layers and twin volume fraction in the Ta layers for Cu/Ta multilayers with NW interface: (a) Cu layer - Shockley, (b) Cu layer - Stair-rod, (c) Cu layer - twinning partial, (d) Ta layer – twin volume fraction.

Supplementary Note 7: damage (voids) distribution for Cu/Ta multilayers with OT interface

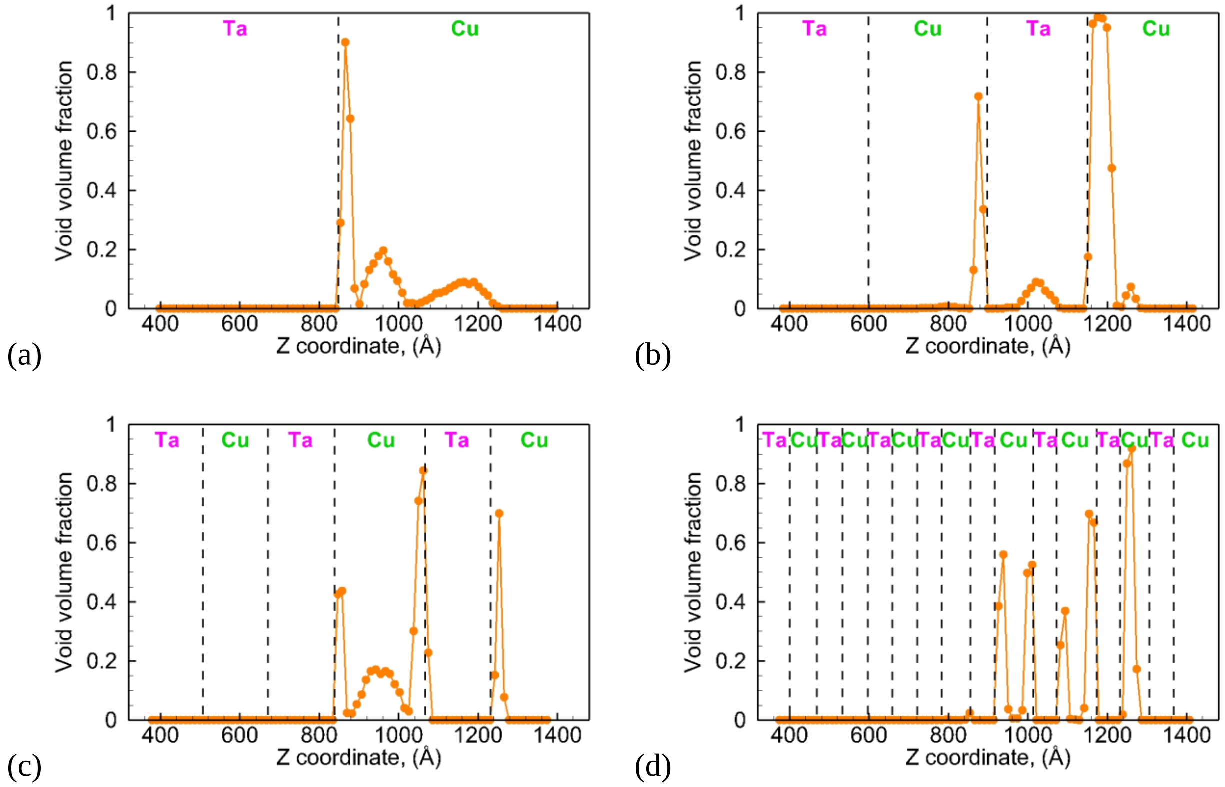

Fig. S5. Distribution of void volume fraction for Cu/Ta multilayers with OT interface at an interface spacing of 16 nm at the time of spall failure (~30 ps): (a) 47 nm, (b) 23 nm, (c) 16 nm, (d) 6 nm. The Cu/Ta interfaces are marked by black dashed lines, and the Cu and Ta layers are marked out.

Supplementary Note 8: spall behavior and dislocation evolution for Cu/Ta multilayers with KS112 interface

Fig. S6 shows the snapshots of the Cu/Ta multilayers with KS112-case1 interface that is representative of KS112 interfaces, and Fig. S7 shows the evolution of dislocation densities in the Cu layers and twin volume fraction in the Ta layers. A transition in the failure mode from the Cu layer interior to Cu/Ta interface is observed as the interface spacing decreases to 6 nm, similar to that observed for the flat interfaces. The variation in the dislocation density and twin volume fraction with interface spacing is quite different. As interface spacing is decreased to 6 nm, a significant decrease in the amounts of twin faults in the Cu layers is observed, as can also be seen from the significantly reduced densities of twinning partials in Fig. S7c. The local densities of twinning partials at the spall plane is much lower, as shown in Table S4, which could contribute to the lower spall strength values at this interface spacing. In addition, due to the suppression of homogeneous dislocation nucleation, the densities of dislocations in the Cu layers increases much slower during SI and SII (Fig. S7a – S7c). On the contrary, the amount of twin in the Ta layers increases as the interface spacing is decreased. Interestingly, at an interface spacing of 6 nm, the twins nucleated in SI and SII do not annihilate during stress relaxation in SIII and SIV (Fig. S7d), resulting in a much higher twin volume fraction in the Ta layers as compared to the other interface spacings and SC-Ta along [112] direction. The increased twin volume fraction at this interface spacing could provide more void nucleation sites at the twin-interface intersections, which could also contribute to a lower spall strength.

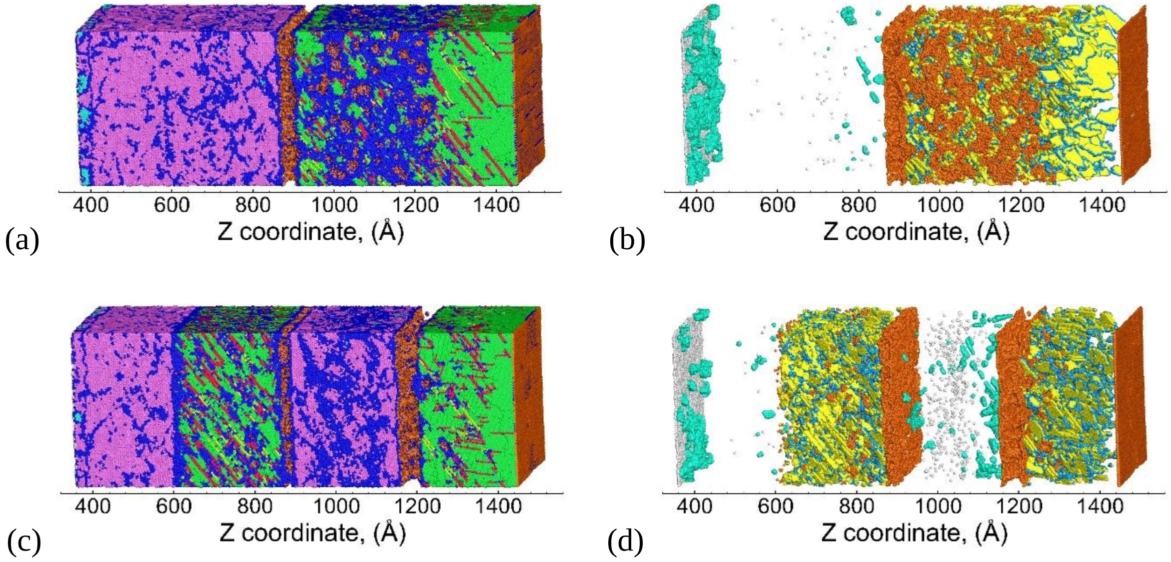

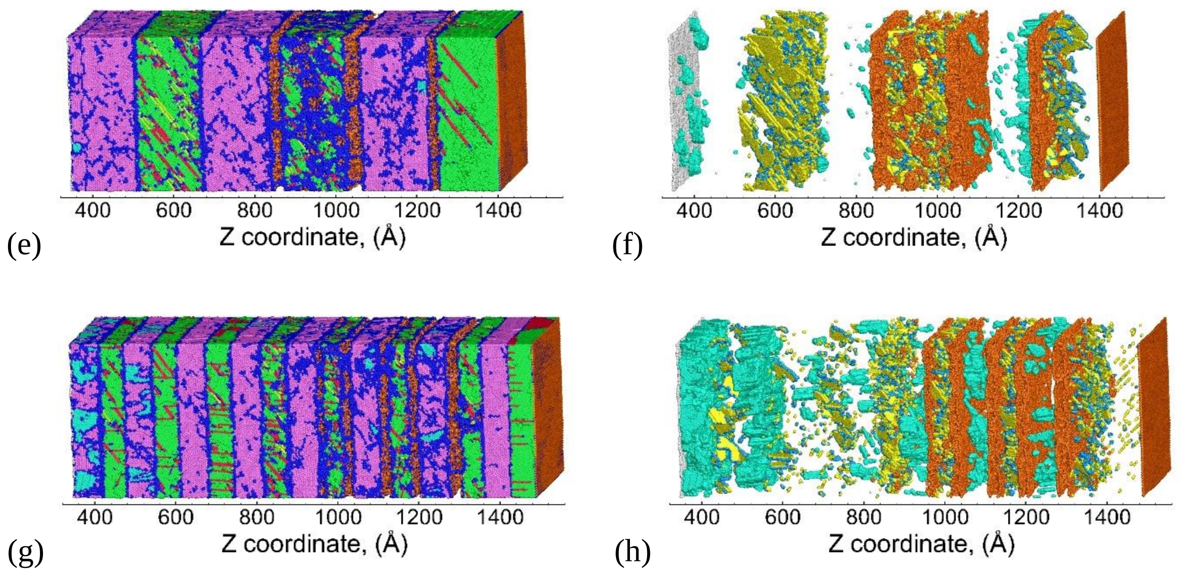

(e) (f) (g) (h)

Fig. S6. Snapshots of Cu/Ta multilayers with KS112-case1 interface at the time of spall failure (~30 ps): (a) – (b) 47 nm, (c) – (d) 23 nm, (e) – (f) 16 nm, (g) – (h) 6 nm. Right-hand panels show the distribution of defects (Cu twin faults, Cu twinning partials, and Ta twin faults) and damage (Cu surfaces and Ta surfaces). Atoms are colored as in Fig. 3.

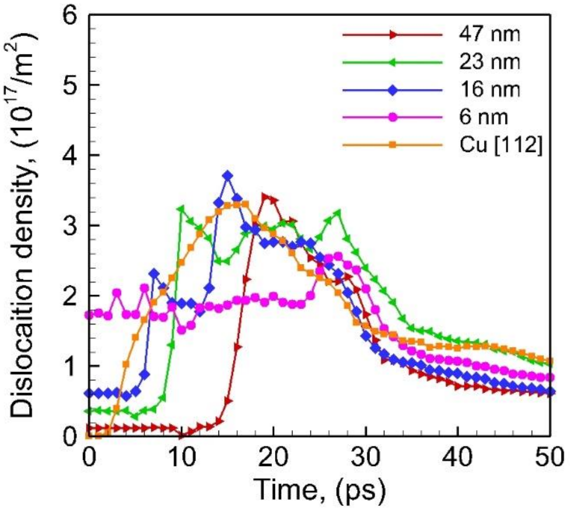

(a) Cu layer – Shockley partial

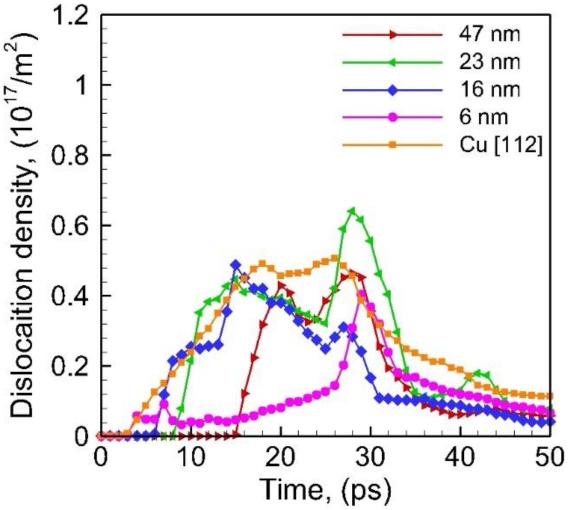

(b) Cu layer – Stairrod partial

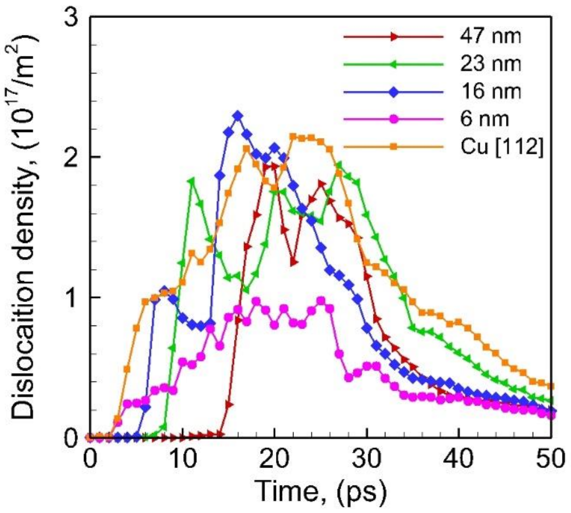

(c) Cu layer – twinning partial

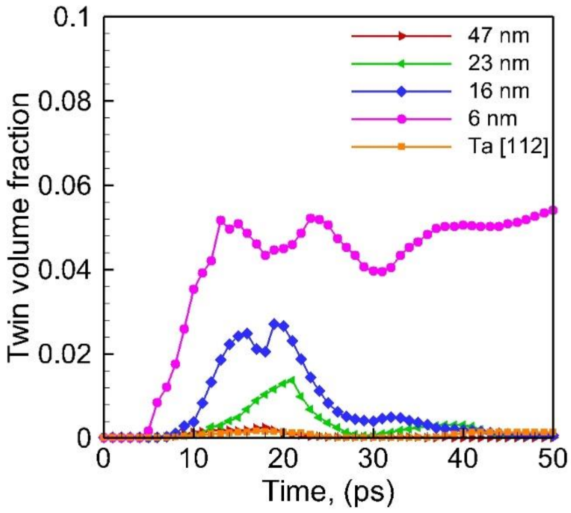

(d) Ta layer – twin volume fraction

Fig. S7. Evolution of overall density of dislocations in the Cu layers and twin volume fraction in the Ta layers for Cu/Ta multilayers with KS112-case1 interface: (a) Cu layer - Shockley, (b) Cu layer - Stair-rod, (c) Cu layer - twinning partial, (d) Ta layer – twin volume fraction.

Supplementary Note 9: dislocation densities in the Cu and Ta layers

Table S4: Dislocation densities ($10^{17}/m^2$) at the spall plane in the Cu layers, density of dislocations with Burgers vector 1/2 <111> and twin volume fraction (f_{twin}) in the Ta layers, peak compressive pressure (P_{max}) and spall strength (σ_{spall}) for the Cu/Ta multilayers, SC-Cu and SC-Ta. The values are taken at a time of peak tensile pressure in the Cu layers (~30 ps).

**Dislocation**		**Cu Layers**			**Ta Layers**		**P_{max} (GPa)**	**σ_{spall} (GPa)**
**Interface**	**λ (nm)**	**Shockley**	**Stair-rod**	**Twin**	**1/2<111>**	**f_{twin}**		
KS	47	1.07	0.20	0.43	1.34	0.019	63.18	10.80
	23	1.12	0.14	0.31	1.12	0.016	72.52	10.70
	16	0.79	0.16	0.29	1.29	0.013	70.69	10.73
	6	0.53	0.09	0.20	1.00	0.001	68.01	10.14
NW	47	1.06	0.19	0.40	1.33	0.020	63.27	10.72
	23	1.12	0.14	0.30	1.15	0.014	71.70	10.68
	16	0.86	0.18	0.31	1.30	0.007	70.59	10.71
	6	0.77	0.14	0.26	1.04	0.002	67.17	10.04
	47	1.09	0.19	0.38	1.39	0.020	63.28	10.64

KS_2	23	1.17	0.17	0.31	1.12	0.010	71.32	10.58
	16	0.78	0.17	0.27	1.29	0.009	70.30	10.86
	6	0.82	0.19	0.34	1.10	0.001	67.69	9.51
KS112-case1	47	1.28	0.26	0.89	1.64	0.006	62.63	10.62
	23	1.19	0.13	0.67	1.13	0.004	73.25	10.98
	16	1.15	0.18	0.62	1.33	0.006	71.43	11.01
	6	1.05	0.17	0.51	0.85	0.049	67.55	10.40
KS112-case 2	47	1.18	0.24	0.76	1.66	0.006	62.47	10.58
	23	1.54	0.18	0.90	1.15	0.003	73.33	10.95
	16	1.11	0.17	0.55	1.23	0.005	71.82	11.02
	6	1.05	0.25	0.46	0.85	0.056	67.93	10.27
OT	47	0.44	0.03	0.28	0.41	0.055	61.92	10.75
	23	0.58	0.03	0.29	0.30	0.030	70.93	10.95*
	16	0.41	0.03	0.18	0.24	0.097	69.55	10.94
	6	0.52	0.03	0.24	0.21	0.135	65.13	10.72
Single-crystal (SC)	SC-Cu [111]	0.69	0.12	0.27	N/A	N/A	44.70	10.06
	SC-Cu [112]	1.28	0.32	1.30	N/A	N/A	55.92	10.76
	SC-Cu [110]	0.90	0.05	0.57	N/A	N/A	63.90	10.74
	SC-Ta [110]	N/A	N/A	N/A	0.83	0.012	78.53	19.48
	SC-Ta [112]	N/A	N/A	N/A	0.94	0.001	80.50	19.88
	SC-Ta [001]	N/A	N/A	N/A	0.16	0.012	83.90	22.75

*Spall is also observed in the Ta layer for this system, with a spall strength of 19.21 GPa.
